# Supplementary figures and images for: Phylogeographic characterization of Burkholderia pseudomallei isolated from Bangladesh
Source: PLoS Negl Trop Dis. 2023 Dec 7;17(12):e0011823. doi: 10.1371/journal.pntd.0011823 (PMC10729972; doi:10.1371/journal.pntd.0011823)

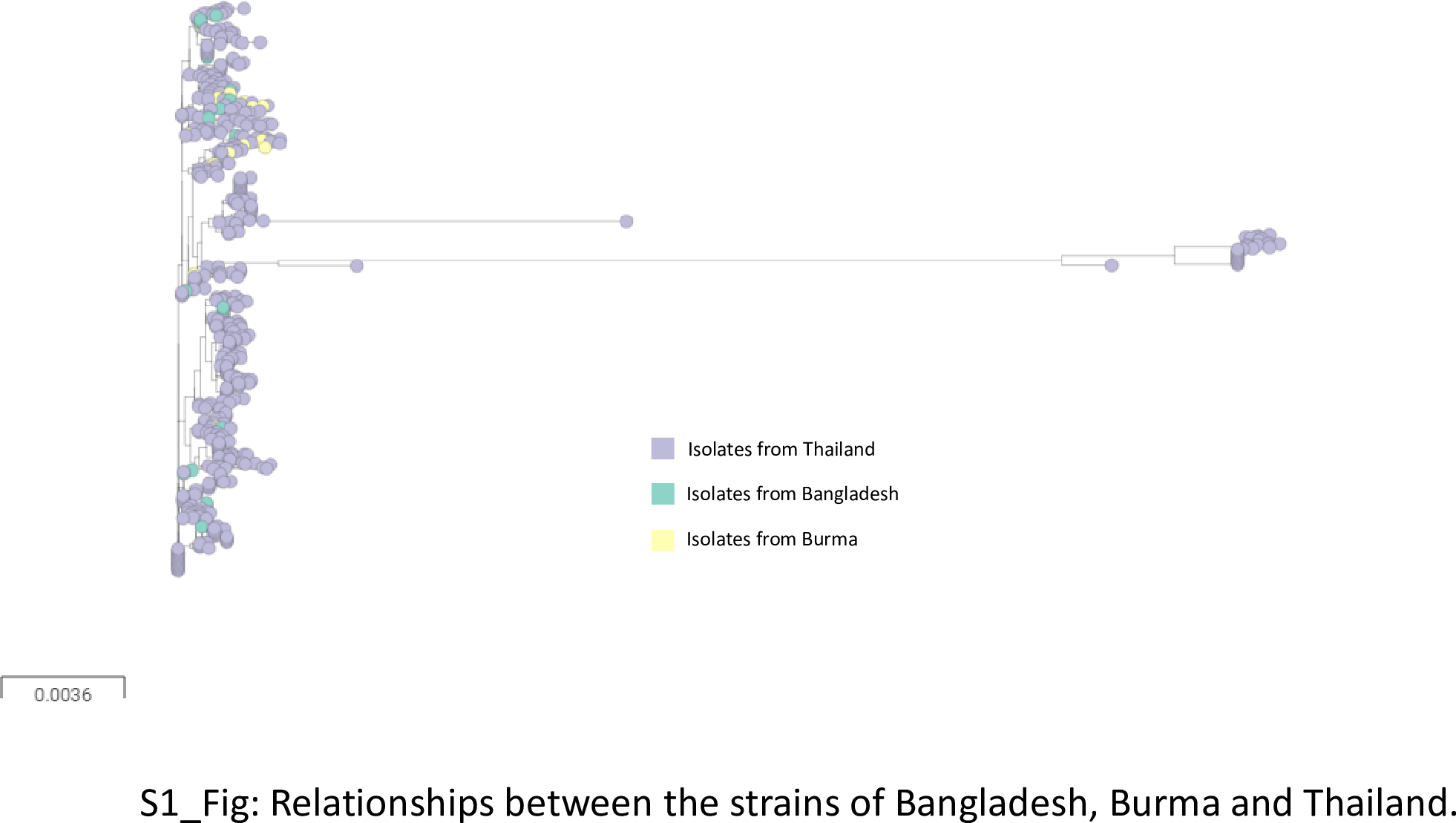

Supplement: S1 Fig — (TIF) [file pntd.0011823.s001.tif]

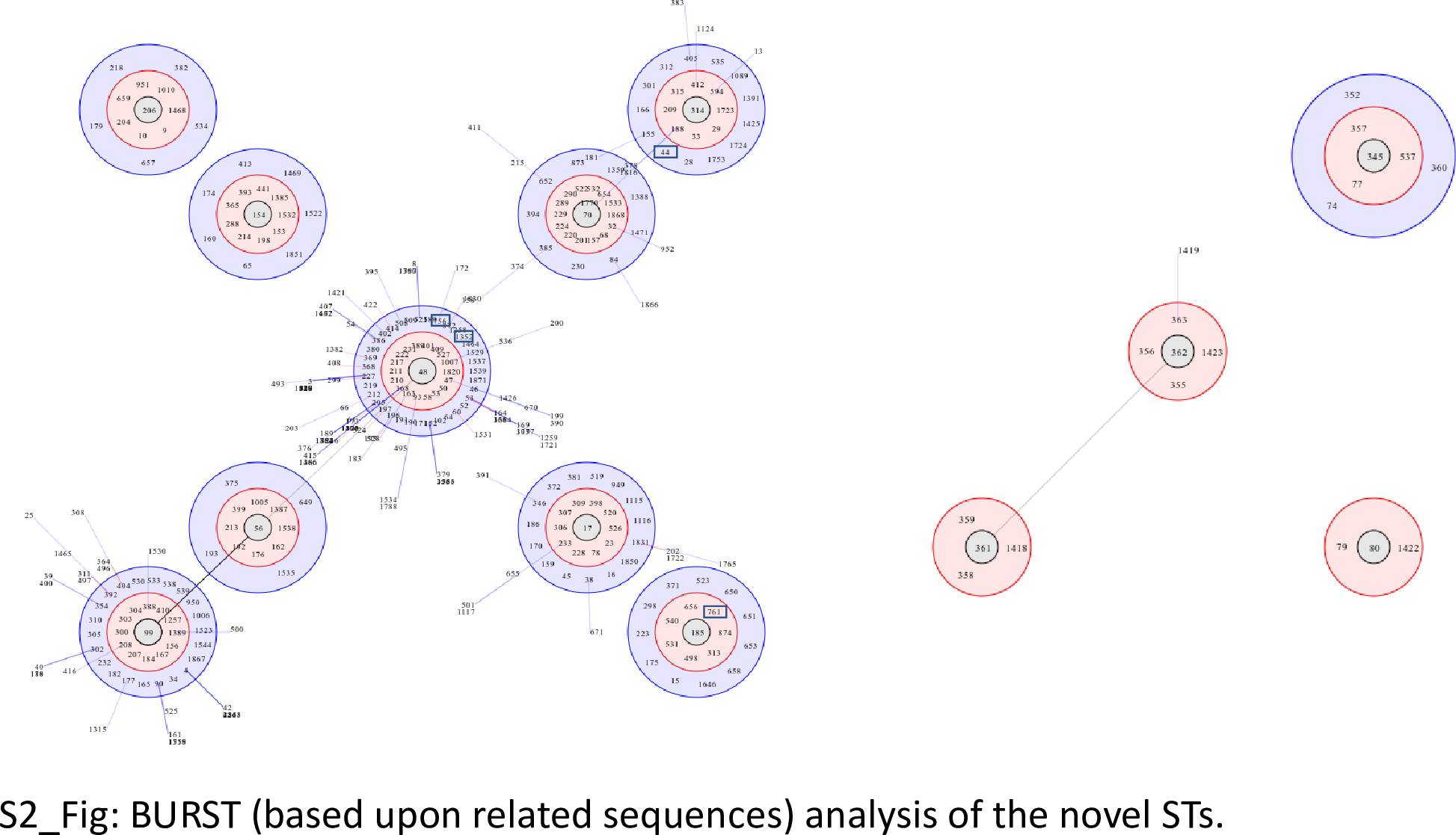

Supplement: S2 Fig — (TIF) [file pntd.0011823.s002.tif]

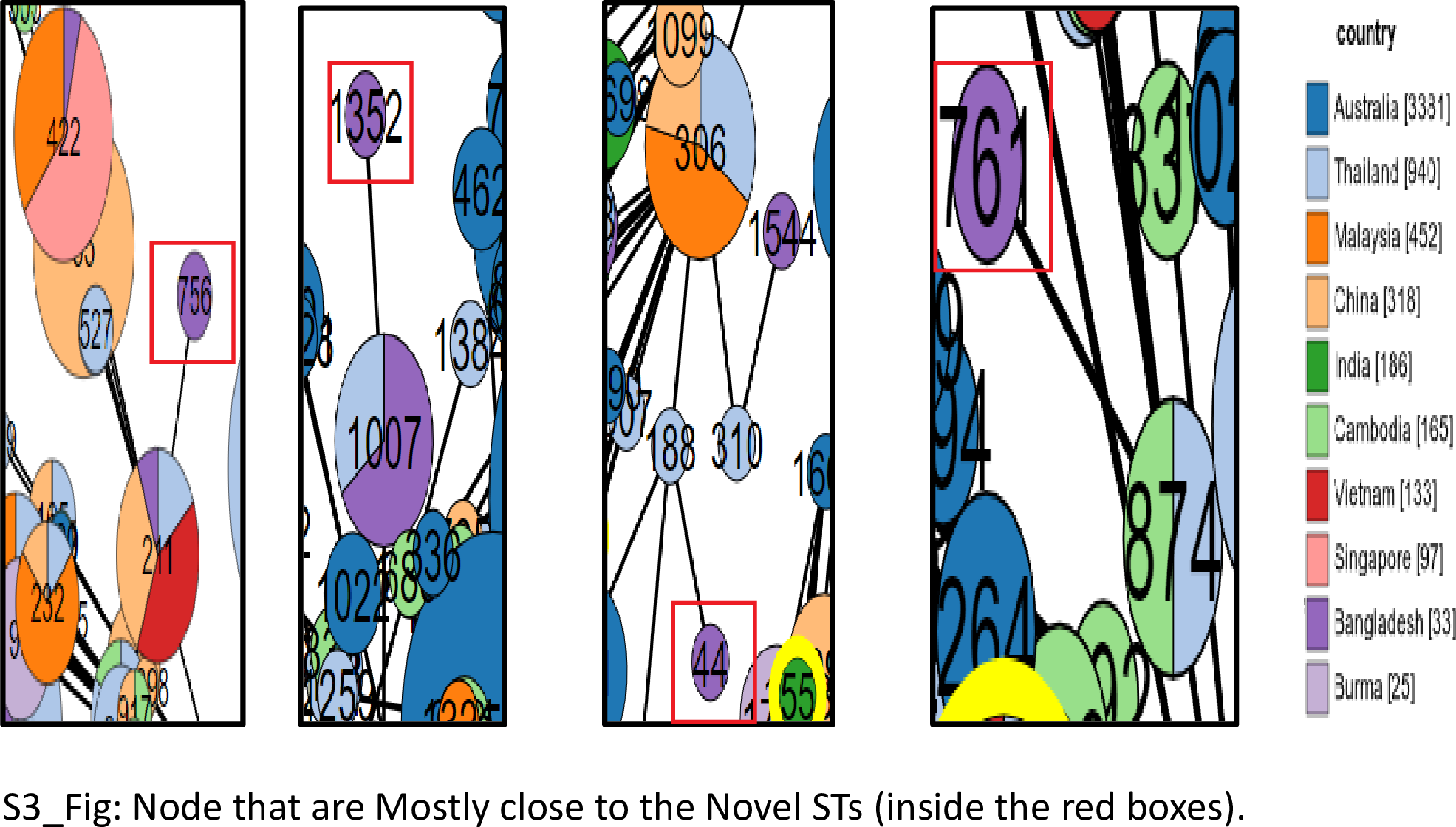

Supplement: S3 Fig — (TIF) [file pntd.0011823.s003.tif]
