# Supplementary material for: Phylogeographic characterization of Burkholderia pseudomallei isolated from Bangladesh
Source: PLoS Negl Trop Dis. 2023 Dec 7;17(12):e0011823. doi: 10.1371/journal.pntd.0011823 (PMC10729972; doi:10.1371/journal.pntd.0011823)
Supplement: Attachment — Submitted filename: PNTD-D-22-00909_Reviewers Report.pdf [file pntd.0011823.s005.pdf]

Reviewer's Report: **Manuscript Number: PNTD-D-22-00909**

Full Title: Phylogeographic characterization of *Burkholderia pseudomallei* isolated from Bangladesh
Short Title: Molecular typing of Burkholderia pseudomallei in Bangladesh
Article Type: Research Article

The Editor,
Plos NTD

Reviewer's General Comments: The Authors attempted to phylogenetic and geographic (phylogeographic) characterization of *Burkholderia pseudomallei* isolated from Bangladesh. Twenty four (24) isolates comprising of 22 clinical and two soil isolates were used in this study where they found a total of 13 different Sequence Type (ST) that includes five novel STs. The study lacked rigorous scientific sampling procedure and spatial spread of sampling to infer phylogeographic distribution of the isolates. You may wish to consider this manuscript as short communication or letter to the Editor if such rooms for publication are available.

Specific Comments with regards to the text of the manuscript are as follows:

S/No.	Section	Lines	Remarks
**1.**	Title		
**2.**	Abstract		Significance: ST 1005 was detected in both soil and clinical samples of Gazipur which indicates the soil to human transmission of this pathogen.<br>***Comments: The organism is already known soil dwelling pathogen***
**3.**			This type of strains may have originated from Vietnam-------<br>***Comments: There are no enough evidences to suggest strain spread from Vietnam to Bangladesh as far as this work is concerned.***
**4.**	Introduction	37-38	Thailand and Singapore, where extensive surveillance had **been** undertaken.<br>***Comments: The authors need to cite some references of those "extensive surveillance" mentioned.***
**5.**		99	----- one reference strain (USM) obtained from Universiti Sans Malaysia.<br>***Comments: Authors need to give the identity of the isolate from USM for reference purpose e.g USM??? Or No..***
**6.**	Methodology	101	------- BIRDEM General Hospital from 2009 to 2015 and two soil strains isolated from-------<br>***Comments: "BIRDEM" if this is an abbreviation it should be written in full the abbreviation in apprentices.***
**7.**		118	MLST was performed according to the methods of Godoy et

			al. ----------<br>***Comments: the citation should follow the PNTD referencing format.***
**8.**		138	Most of the patients came from Gazipur and Tangail.<br>***Comments: Since the work is solely on Phylogeography, the authors need to give location-specific prevalence of the 22 isolates.***
**9.**		166-167	ST 300 has been found in Thailand, India and Myanmar.<br>***Comments: ST should be in full at the beginning of sentence.***
**10.**		172	Supplementary Figure 1<br>***Comments: Why not Figure 1 instead of Supplementary Figure 1.Since subsequent figures in 174 is referred to Figure 2***
**11.**		174	ST 56 is predominant in both Bangladesh and Myanmar<br>***Comments: ST should be in full at the beginning of sentence.***
**12.**		188	ST 1352 clustered with the clade of--------<br>***Comments: Same as 174***
**13.**		189	ST756 did not cluster in any clades, however, demonstrated close relation with--------<br>***Comments: Same as 174***
**14.**		192-194	Supplementary Figure 2 and Supplementary Figure 1, including Supplementary Figure 1 were not among the figures presented in the manuscript. Since they are part of the results presented they should be included.
**15.**	Discussions	219-222	Comments: This section is a repetition of the results presentation in discussion.
**16.**		222	ST 1005, ST 56, ST 1007 and ST 211 have been detected not only in Bangladesh-------------<br>***Comments: Same as 174***
**17.**		225	ST 56 which was present in five clinical 225 isolates is------<br>***Comments: Same as 174***
**18.**		264-268	An important feature of melioidosis is the spatial clustering of clinical incidence which is linked to the environmental prevalence of the organism-------<br>***Comments: This section is not relevant to this work as no environmental samples were collected to correlate with soil types***
**19.**		275-277	B. pseudomallei possessing ST 46 was recovered in 1960 from a crewman travelling through----------<br>***Comments: This section is redundant as this work did not recovered ST46.***
**20.**		283-286	It is likely that human and/or animal movements between these areas--------<br>***Comments: This section is irrelevant and redundant as no animal isolates are involved in this study nor animal movements***
