# Supplementary material for: Phylogeographic characterization of Burkholderia pseudomallei isolated from Bangladesh
Source: PLoS Negl Trop Dis. 2023 Dec 7;17(12):e0011823. doi: 10.1371/journal.pntd.0011823 (PMC10729972; doi:10.1371/journal.pntd.0011823)
Supplement: Attachment — Submitted filename: PNTD-D-22-00909_reviewer1.pdf [file pntd.0011823.s006.pdf]

PLOS Neglected Tropical Diseases

Phylogeographic characterization of Burkholderia pseudomallei isolated from Bangladesh

--Manuscript Draft--

**Manuscript Number:**	PNTD-D-22-00909
**Full Title:**	Phylogeographic characterization of Burkholderia pseudomallei isolated from Bangladesh
**Short Title:**	Molecular typing of Burkholderia pseudomallei in Bangladesh
**Article Type:**	Research Article
**Keywords:**	Melioidosis, Burkholderia pseudomallei, MLST, phylogeography
**Abstract:**	Background: Burkholderia pseudomallei possesses a diverse set of genes which encode a vast array of biological functions reflecting its clinical, ecological and phenotypic diversity. Strain variation is linked to geographic location as well as pattern of land uses. This soil-dwelling Gram-negative pathogen causes melioidosis, a tropical disease endemic in northern Australia and Southeast Asian regions including Bangladesh. Phylogeographic analyses of B. pseudomallei isolates by molecular typing techniques could be used to examine the diversity of this organism as well as to track melioidosis epidemics.<br>Methods: In this study, 24 B. pseudomallei isolates, of which 22 clinical and two soil isolates were analyzed, utilizing Real-time PCR assay and multilocus sequence typing (MLST). The sequences were then submitted to PubMLST database for analysis and construction of phylogenetic tree.<br>Findings: A total of 13 different Sequence Type (ST) that includes five novel STs were identified for the first time. Strains having ST 1005, 1007 and 56 were the most widespread STs frequently isolated in Bangladesh. ST 1005, ST 56, ST 1007 and ST 211 have been detected not only in Bangladesh but are also present in many Southeast Asian countries.<br>Significance: ST 1005 was detected in both soil and clinical samples of Gazipur which indicates the soil to human transmission of this pathogen. Most prevalent, ST 56 has been previously reported from Myanmar, Thailand, Cambodia and Vietnam, confirming the persistence of the strain over the entire continent. This type of strains may have originated from Vietnam and spread via Myanmar to Bangladesh but further identification of isolates carrying ST 56 are required to confirm this hypothesis. Further large-scale study is necessary to find out the magnitude of the infection and its different reservoirs in the environment along with phylogeographic association.
**Additional Information:**	
**Question**	**Response**
**Financial Disclosure**<br><br>Enter a financial disclosure statement that describes the sources of funding for the work included in this submission. Review the submission guidelines for detailed requirements. View published research articles from *PLOS NTDs* for specific examples.<br><br>This statement is required for submission and **will appear in the published article** if the submission is accepted. Please make sure it is accurate.	The author(s) received no specific funding for this work.

Powered by Editorial Manager® and ProduXion Manager® from Aries Systems Corporation

**Unfunded studies**<br><br>Enter: *The author(s) received no specific funding for this work.*<br><br>**Funded studies**<br><br>Enter a statement with the following details:<br>• Initials of the authors who received each award<br>• Grant numbers awarded to each author<br>• The full name of each funder<br>• URL of each funder website<br>• Did the sponsors or funders play any role in the study design, data collection and analysis, decision to publish, or preparation of the manuscript?<br>• **NO** - Include this sentence at the end of your statement: *The funders had no role in study design, data collection and analysis, decision to publish, or preparation of the manuscript.*<br>• **YES** - Specify the role(s) played.<br><br>* typeset	
**Competing Interests**<br><br>Use the instructions below to enter a competing interest statement for this submission. On behalf of all authors, disclose any competing interests that could be perceived to bias this work—acknowledging all financial support and any other relevant financial or non-financial competing interests.<br><br>This statement **will appear in the published article** if the submission is accepted. Please make sure it is accurate. View published research articles from *PLOS NTDs* for specific examples.	The authors have declared that no competing interests exist.

**NO authors have competing interests**<br><br>Enter: *The authors have declared that no competing interests exist.*<br><br>**Authors with competing interests**<br><br>Enter competing interest details beginning with this statement:<br><br>*I have read the journal's policy and the authors of this manuscript have the following competing interests: [insert competing interests here]*<br><br>* typeset<br><br>This statement is required for submission and **will appear in the published article** if the submission is accepted. Please make sure it is accurate and that any funding sources listed in your Funding Information later in the submission form are also declared in your Financial Disclosure statement.	
**Data Availability**<br><br>Authors are required to make all data underlying the findings described fully available, without restriction, and from the time of publication. PLOS allows rare exceptions to address legal and ethical concerns. See the PLOS Data Policy and FAQ for detailed information.	Yes - all data are fully available without restriction

A Data Availability Statement describing where the data can be found is required at submission. Your answers to this question constitute the Data Availability Statement and **will be published in the article**, if accepted.<br><br>**Important:** Stating 'data available on request from the author' is not sufficient. If your data are only available upon request, select 'No' for the first question and explain your exceptional situation in the text box.<br><br>Do the authors confirm that all data underlying the findings described in their manuscript are fully available without restriction?	
**Describe where the data may be found in full sentences. If you are copying our sample text, replace any instances of XXX with the appropriate details.**<br><br>• If the data are **held or will be held in a public repository**, include URLs, accession numbers or DOIs. If this information will only be available after acceptance, indicate this by ticking the box below. For example: *All XXX files are available from the XXX database (accession number(s) XXX, XXX.*).<br>• If the data are all contained **within the manuscript and/or Supporting Information files**, enter the following: *All relevant data are within the manuscript and its Supporting Information files.*<br>• If neither of these applies but you are able to provide **details of access elsewhere**, with or without limitations, please do so. For example:	Not applicable

Data cannot be shared publicly because of [XXX]. Data are available from the XXX Institutional Data Access / Ethics Committee (contact via XXX) for researchers who meet the criteria for access to confidential data.<br><br>*The data underlying the results presented in the study are available from (include the name of the third party and contact information or URL).*<br>• This text is appropriate if the data are owned by a third party and authors do not have permission to share the data.<br><br>* typeset	
Additional data availability information:	

Powered by Editorial Manager® and ProduXion Manager® from Aries Systems Corporation

Manuscript
Click here to access/download;Manuscript;MLST Manuscript.docx

Title: Phylogeographic characterization of *Burkholderia pseudomallei* isolated from Bangladesh

1. **Md. Shariful Alam Jilani***, PhD, *Department of Microbiology, Ibrahim Medical College, Dhaka, Bangladesh.*
2. **Saika Farook**, MD, *Department of Microbiology, Ibrahim Medical College, Dhaka, Bangladesh.*
3. **Arittra Bhattacharjee**, MSc, *Bioinformatics Division, National Institute of Biotechnology, Savar, Dhaka, Bangladesh*.
4. **Lovely Barai**, PhD, *Department of Microbiology, BIRDEM* General Hospital, *Dhaka, Bangladesh*.
5. **Chowdhury Rafiqul Ahsan**, PhD, *Department of Microbiology, University of Dhaka, Dhaka, Bangladesh*.
6. **Jalaluddin Ashraful Haq**, PhD, *Department of Microbiology, Ibrahim Medical College, Dhaka, Bangladesh.*
7. **Apichai Tuanyok,** PhD, *Department of Infectious Diseases and Immunology, College of Veterinary Medicine; University of Florida, Florida, USA.*

***Correspondence**

Md. Shariful Alam Jilani

Professor and Head

Department of Microbiology

Address: 1/A Ibrahim Sarani, Segun Bagicha, Dhaka-1000, Bangladesh.

Email: jilanimsa@gmail.com

Phone number: +8801712146927

Introduction

The soil-dwelling bacterium *Burkholderia pseudomallei* is responsible for causing the deadly disease melioidosis. Once considered an obscurity, the disease is now recognized as an emerging fulminating infectious disease of global significance. The hyper-endemic foci of this disease exist in northern Australia, Thailand and Singapore, where extensive surveillance had undertaken. Since melioidosis is a disease of rural poor and usually affects the people in the area that are poorly supplied with the diagnostic capability to make the diagnosis, so the true burden of this disease throughout the globe remains abstruse [1]. However, in recent years melioidosis has been increasingly reported from diverse tropical regions of the globe where it has not been detected previously [2-4]. It is not clear whether the spread of melioidosis beyond the known endemic regions has occurred in recent decades, or it is just being unveiled by better recognition, increased awareness and surveillance. It has been hypothesized that *B. pseudomallei* may have originated in Australia and subsequently spread to South and Southeast Asia via animal migration during the Miocene period about 15 million years ago when the continents were joined by a land bridge due to greatly lowered sea levels [5]. From Asia *B. pseudomallei* eventually migrated to Madagascar and Africa and then through slave trading from West African Countries to America [6,7].

The genome of *B. pseudomallei* encompasses a diverse set of genes which encode a vast array of biological functions reflecting its clinical, ecological and phenotypic diversity. Strain variation among *B. pseudomallei* is linked to geographic location as well as pattern of land uses [8]. It has

been determined that Australian isolates usually possess an ancestral *B. thailandensis*-like flagellum and chemotaxis (BTFC) gene cluster, whilst isolates from Asia almost exclusively carry a *Yersinia*-like fimbrial (YLF) gene cluster [9-10]. *B. pseudomallei* strains containing the YLF gene cluster are associated with clinical cases, whereas strains containing the BTFC gene clusters are more likely to be environmental isolates, indicating a potential role for the YLF gene associated with virulence [8]. This variation in clinical manifestations between melioidosis in Australia and Southeast Asia suggests that these populations are genetically distinct due to broad-scale bio-geographical factors associated with the establishment and persistence of the organism. Melioidosis is caused by contact with contaminated surface water or soil, with human-to-human transmission being exceptionally rare. Thus, large geographic barriers, as for example, the Wallace Line, which separates most of Asia from Australia, have subsequently restricted gene flow between populations. These factors have led to distinct Asian and Australasian *B. pseudomallei* populations that have regionally evolved over hundreds or thousands of years. However, recent whole-genome sequencing has resolved that Asian isolates of *B. pseudomallei* share an Australian ancestral root [11].

Melioidosis has been sporadically detected in Bangladesh over last several decades [12]. Till 2021, a total of 86 culture confirmed *B. pseudomallei* has been isolated from 68 melioidosis cases [13]. Although, the first case of melioidosis from Bangladesh is believed to be identified in a 29-year-old British sailor in 1960, the persistence of the organism in soil was first confirmed 52 years later in 2012 [14,15]. *B. pseudomallei* was isolated for the first time in Indian Subcontinent from environmental samples from Gazipur district of Bangladesh. Since then, Bangladesh is considered as a definitive country for melioidosis [15]. However, the true burden

of the disease as well as mortality rates attributed to melioidosis is unknown, due to a lack of extensive epidemiological surveillance program. For that reason, molecular analyses of *B. pseudomallei* isolates by multilocus sequence typing (MLST) may be used to examine the diversity of this organism from various geographic regions. This will assist to assess population structure of the organism as well as form phylogeographic characteristics that can be used to track melioidosis epidemics [16].

The current study demonstrates the utility of the MLST scheme for epidemiological studies and clarifies the genetic relationships between *B. pseudomallei* isolated from the clinical specimen and environmental sources. Understanding the population genetic structure and existing biodiversity of *B. pseudomallei* in endemic environments has implications for tracing outbreak source [17]. Detailed phylogenetic study of isolates will advance understanding of the biogeography of *B. pseudomallei* from this region as well as from the neighboring countries. In the present study, we examined the strains isolated from both clinical specimen and soil samples from different areas of Bangladesh using microbiological and molecular tests. We then compared the MLST data with those of the *B. pseudomallei* MLST database to determine the genetic relatedness and attempted to provide an insight into the ecology of *B. pseudomallei* through an elucidation of the mechanisms involved in evolution, speciation, and dispersal.

Methodology

Ethics Statement

The Ethical Review Committee (ERC) of the Diabetic Association of Bangladesh (BADAS) has approved the study. Ibrahim Medical College (IMC) is an institution under the BADAS, where the study was conducted.

Sample collection and isolation of Bacterial strains

The study utilized a total of 25 *B. pseudomallei* isolates that included 22 clinical, two soil isolates and one reference strain (USM) obtained from Universiti Sans Malaysia. The study availed archived clinical isolates, which were obtained from diabetic patients who attended BIRDEM General Hospital from 2009 to 2015 and two soil strains isolated from Kapasia, Gazipur as previously described [12,15]. The sample collecting regions of Bangladesh has been demonstrated in Figure 1 using DIVA-GIS (www.diva-gis.org). Bacteria were stored in Trypticase Soy Broth supplemented with 15% glycerol. Isolates were plated onto Ashdown's agar and cultivated at 37°C for 48 hours before DNA extraction [18]. Real-time PCR assay followed by MLST from extracted DNA was performed at the Emerging Pathogens Institute, University of Florida, USA.

Real-time PCR assay

From the extracted DNA Real-time PCR assay was done for molecular identification of Type III secretion system 1 (TTS1). Primer pair and probe were used to target the TTS1 gene cluster of *B. pseudomallei* as previously described [19]. The YLF and BTFC is a multiplex real-time PCR assay using SYBR green as a fluorescent dye targeting YLF and BTFC gene cluster developed

by Tuanyok A et al in 2007. PCR primers for detecting YLF and BTFC genes were used as previously described [9].

Multilocus sequence typing (MLST) of the bacterial strains

MLST was performed according to the methods of Godoy et al by amplifying and sequencing of the seven housekeeping gene fragments (*ace*, *gltB*, *gmhD*, *lepA*, *lipA*, *narK* and *ndh*).The sequences were submitted to the PubMLST database (https://pubmlst.org/) for further analysis [20,21]. The presence of the Sequence type (ST) in organisms detected in other countries was identified by exploring the PubMLST datasets for *B. pseudomallei*. Genetic relations between the isolates from Bangladesh and other countries that have found the same ST were visualized using GrapeTree and Microreact [22,23].

Characterization of the New ST

To characterize the genomic makeup of the new ST, the nucleotide sequences of the selected housekeeping genes were taken to construct a neighbor-joining (NJ) based phylogenetic tree with the ST of Bangladesh [24]. The tree was constructed via PubMLST and visualized by iTOL [25]. After that the Based upon Related Sequences (BURST) algorithm was applied on the isolates of Bangladesh, Myanmar and Thailand to identify the relations of the genotypes in major clonal clusters with default group definition. The central ST of a BURST group have the highest number of single locus variants (SLVs). Generally, the most frequent ST take the central roles in the cluster/clonal complex and therefore helps to characterize the genotypic behaviors of the new ST [26,27].

Result

Isolates were collected from melioidosis patients and soil

A total of 24 *B. pseudomallei* isolates were collected of which 22 were clinical, and two were environmental, from 2009 to 2015. Most of the patients came from Gazipur and Tangail. However, only one patient reported from Khagrachari which is geographically closer to Southeast Asian country Myanmar (Figure 1). Both isolates from soil samples were obtained from Gazipur [15].

Real-time PCR identified all isolates as YLF positive *B. pseudomallei*

Real-time PCR assay, targeting TTS1, confirmed that all 22 clinical and two environmental isolates were *B. pseudomallei* along with the USM reference strain. All isolates contain YLF gene cluster and are negative to BTFC gene cluster.

Figure 1: Geographical locations of the melioidosis cases.

MLST profiling of *B. pseudomallei* from Bangladesh

MLST unveiled a total of 13 ST of which five ST (ST- 756, 1352, 761, 44 and 61) were novel types and identified for the first time (Table 1). Strains having ST 1005, 1007 and 56 were the most frequently isolated ST in Bangladesh. ST 1005, ST 56, ST 1007 and ST 211 have been detected not only in Bangladesh but are also present in Thailand, Cambodia, Australia, Myanmar, China and Vietnam. Previously all the *B. pseudomallei* ST 1005 were collected from the environmental samples of Thailand and Australia. Even in our study, we obtained two *B. pseudomallei* ST 1005 from the soil of Gazipur. However, according to PubMLST database we found, for the first time, two isolates with ST 1005 from clinical samples of humans residing in the same district.

Table 1: Sequence type (ST) of *B. pseudomallei* in the present study. ST that were common in other countries are also given.

Isolates reported from Bangladesh				No. of strains listed on PubMLST database	ST reported from Bangladesh and other geographic locations
**ST**	**No. of isolates**	**Clinical**	**Environmental**		
1005	4	2	2	25	Thailand, Australia
56	5	5	0	18	Myanmar, Thailand, Cambodia & Vietnam
756	2	2	0	2	*Only in Bangladesh*
1007	4	4	0	6	Thailand
211	1	1	0	14	Thailand, China, Vietnam
172	1	1	0	2	Thailand
300	1	1	0	9	Thailand, India, Myanmar
422	1	1	0	17	Singapore, Malaysia
1352	1	1	0	1	*Only in Bangladesh*
761	1	1	0	1	*Only in Bangladesh*
1124	1	1	0	1	India
61	1	1	0	1	*Only in Bangladesh* (Expatriate in Brunei)
44	1	1	0	1	*Only in Bangladesh*

ST= Sequence type

Predominant ST of Bangladesh share more genetic similarities with Southeast Asian strains rather than South Asian

Relationships between the ST were determined by GrapeTree (Figure 2). GrapeTree implemented a novel minimum spanning tree algorithm to rebuild these relationships [22]. Most of the isolates of this study are connected genetically to ST 300. ST 300 has been found in Thailand, India and Myanmar. Relatives of ST 300 such as ST 56, 1005 and 1007 are predominant in Bangladesh. These ST have also been reported in Myanmar, Thailand, Cambodia, Vietnam and Australia. However, none of these ST were found in the neighboring country India.

Microreact revealed close distance among the isolates of Myanmar, Thailand and Bangladesh via NJbased tree (Supplementary Figure 1). Two field breakdowns of the dataset revealed the

frequencies and the mutual presence of ST 56 and ST 300 in Myanmar, Thailand and Bangladesh. ST 56 is predominant in both Bangladesh and Myanmar (Figure 2).

Figure 2: Genetic relationships of *B. pseudomallei* isolates between the countries that share common sequence types with Bangladesh. Most of the sequence types (ST) of Bangladesh are connected to ST300 (Labeled in red box). Relatives of ST-300 such as ST-56, 1005 and 1007 are mostly prevalent in Bangladesh.

Figure 3: Bangladeshi strains of *B. pseudomallei* share more genetic similarities with Southeast Asian strains rather than South Asian isolates. The common *B. pseudomallei* strains among Bangladesh, Burma (Myanmar) and Thailand are ST56 and ST300. In the pointed regions (labeled with red arrows) ST that are closely related to ST300 have shown higher prevalence. ST56, a closely related member of ST300, also showed predominance of melioidosis in Bangladesh.

Novel ST are also closely related to Southeast Asian genotypes (especially Thailand)

Five novel genotypes (ST-1352, 44,756, 61 and 761) were found in this study. Among the ST of Bangladesh, ST-44 and 761 clustered in clade A (Figure 4). ST 1352 clustered with the clade of ST-1007 and 46. ST756 did not cluster in any clades, however, demonstrated close relation with clade B (Figure 4). Members of the same clades share more genetic similarities than distant clades. ST-1352, 44, 756 and 761were closely related with ST-1007, 188, 211, 874, respectively (Supplementary Figure 3). BURST analysis depicted that ST-1352 and 756 are double locus variant (DLV) of ST 48. ST 44 is a DLV of ST 314, while ST 761 is a single locus variant (SLV) of ST 185 (Supplementary Figure 2). All these central genotypes were found in Thailand or other Southeast Asian countries such as Vietnam and China.

Figure 4: Phylogenetic relationships of *B. pseudomallei* in Bangladesh. On the right side, the columns depict the ID of the strain in the PubMLST database, sequence type (ST), specimen, source of the isolate and year of isolation. Here, some of the isolates have clustered together and generated clades A, B and C.

Discussion

Melioidosis, a severe disease caused by *B. pseudomallei,* is increasingly recognized in tropical and subtropical areas worldwide. In recent years, greater awareness among clinicians and

microbiologists along with better laboratory facilities in Bangladesh aided in the isolation of the organism in growing numbers thus necessitating the understanding of population structure of the pathogen.

Real Time-PCR assay targeting both YLF and BTFC gene cluster in our clinical and soil isolates detected the presence of only YLF gene cluster in Bangladesh. Studies have demonstrated that *B. pseudomallei* evolved in Australia and subsequently spread to Asia during the last glacial period [28]. Through this spreading, some isolates gained YLF gene cluster and others acquired BTFC gene cluster via horizontal gene transfer. The YLF group predominates in Asia while BTFC group is more prevalent in Australia. It is interesting to note that the YLF group is more common in clinical cases whereas BTFC group is more frequent in environmental isolates [9]. In Thailand, where YLF is dominant, mortality rates for the primary disease are higher (50%) than the endemic regions of Australia, where mortality rate is around 15 to 20% [29,30]. In an earlier study conducted in Bangladesh, sero-positivity rate among healthy individual was found to be 22·6%-30·8% [15]. If reactivation occurs in these cases due to an immune-compromised state, the YLF dominance may play a vital role in elevating the mortality rates.

A total of 13 different sequence types (ST) was found by MLST. The phylogenetic tree showed two groups of ST. One group belongs to ST 56 while another group (non-ST 56) has high sequence diversity (Figure 4). This sequence diversity indicates the endemicity of the pathogen [6,31]. ST 1005, ST 56, ST 1007 and ST 211 have been detected not only in Bangladesh but are also present in Thailand, Cambodia, Australia, Myanmar, China and Vietnam. Additionally, *B. pseudomallei* ST 42, 43, 46, and 71 were previously identified from Bangladeshi patients [20].

ST 56 which was present in five clinical isolates is the most common variant present in Bangladesh, followed by ST 1005 and ST 1007.

According to PubMLST, at least five ST (ST-1352, 44, 756, 761 and 61) were novel types and identified for the first time. It can be assumed that the existence of novel genotypes point towards local persistence of *B. pseudomallei* in the same geographical area and their ability to establish a new clone series producing novel offspring that carry new genotypes [32]. Most of these novel ST were single locus variant and double locus variant of strains from Thailand, which indicate the clonal expansion of their ancestors in this region were due to endemicity.

MLST based molecular surveillance underlined a strong genetic relationship among Chinese, Indian, Myanmar and Southeast Asian *B. pseudomallei* isolates [6,31-34]. All of these studies found common ST with that of strains isolated from Thailand where melioidosis is widespread. Apparently, similar pattern was also observed after MLST profiling in our isolates. The genetic relationship of all the available ST from Bangladesh was visualized via GrapeTree. All of the isolates are genetically related with ST 300 and ST 354, which have been found in Southeast Asian regions (Figure 3). ST 300 and ST 56 are also isolated from neighboring countries, Myanmar and Thailand. In addition, other ST like ST 172, ST 211, ST 1005 and ST 1007 share more genetic similarities with Southeast Asian strains than South Asian isolates. Only, ST 1124 and ST 300 were common between India and Bangladesh. Therefore, most plausibly, the major introduction of *B. pseudomallei* took place via Southeast Asian regions in Bangladesh. This observed relatedness may be explained by *B. pseudomallei* transmission into Bangladesh due to geographical propinquity to neighbors, human and animal migration, ancient and persisting trading and travel networks. Previous studies demonstrated that Malay Peninsula was repeatedly

reintroduced with *B. pseudomallei* [6,28]. However, to identify this type of reintroductions in Bangladesh, comparative genomics-based investigations are necessary.

The most common variant (i.e., ST 56) found in Bangladesh, was isolated from patients of diverse regions of the country. ST 56 was also isolated from a patient in 1983, as per PubMLST database, which suggests the existence of the strain in the country for over a period of 32 years. ST 56 has been previously reported from Myanmar, Thailand, Cambodia and Vietnam, confirming the persistence of the strain, causing infection over the entire continent. This type of strains may have originated from Vietnam and spread via Myanmar to Bangladesh but further identification of isolates carrying ST 56 are required to confirm this hypothesis [6]. However, our ST 56 isolates are suspected to be highly virulent since all five of them were isolated from patients' blood (Supplementary Table-1). Two out of these five septicemic patients died. The first melioidosis case was detected in a Bangladeshi infant, suffering from pneumonia and septicemia, who died 12 hours following hospital admission [35]. This case was probably identified as the same ST 56 case in accordance with PubMLST data, which leads us to suspect that some distinct virulence factors are associated with this specific sequence type in Bangladesh. However, whole genome sequencing followed by in vivo and in vitro validations are required to provide clear pathophysiology.

An important feature of melioidosis is the spatial clustering of clinical incidence which is linked to the environmental prevalence of the organism. The presence of *B. pseudomallei* is higher in anthrosol than acrisol soil types. Anthrosol is a soil type that has been highly modified by human for the preparation of paddy fields whereas acrisol is enriched with clay-rich soil common in tropical climates [36]. We found two ST 1005 isolates from anthrosol type of soil from Gazipur.

ST 1005 was also isolated from clinical samples of Gazipur. These are the first clinical cases of ST 1005 according to PubMLST database. This strongly indicates that spatial clustering of clinical incidence is linked to the environmental persistence of the organism and infection follows environmental exposure. In addition, this may also point out that naturally occurring groundwater functions as a vehicle for the dispersal of viable *B. pseudomallei* away from a primary environmental reservoir.

B. pseudomallei possessing ST 46 was recovered in 1960 from a crewman travelling through Bangladesh and also from a monkey in Indonesia; however, this ST has not been recovered in any patient in the current study [20]. The presence of ST 46 in both humans and animals indicates that the same clone of *B. pseudomallei* appears to be able to cause melioidosis in both humans and animals. Most examples of the same ST recovered from different species with the disease is likely to be due to the independent acquisition of the same virulent strain from the environment, although direct transmission from infected animals to humans has been proposed [36]. It is to be mentioned that *B. pseudomallei* isolated earlier from Bangladeshi individuals living abroad reported to contain ST 42, 43, 46, 56 and 71 [20]. It is likely that human and/or animal movements between these areas played pivotal role in the dissemination of *B. pseudomallei*, however analysis of broader range of isolates from this region are required for confirmation.

The detection of different sequence types in a defined area indicates the historical introduction and dissemination of different genotypes into the area or due to expansion of local ST that yielded new strains with novel ST [32]. MLST is an unambiguous and powerful tool to study the bacterial populations and its global distribution [20]. However, MLST is not very much efficient

in detecting relatedness among *B. pseudomallei* ST due to high levels of lateral gene transfer in *B. pseudomallei* strain. In order to determine the origin of the organism in Bangladesh, its introduction and dissemination to other area and evolutionary change among strains require a whole genome-based single nucleotide polymorphism typing. Such study will also help us to understand the phylogeographic association of *B. pseudomallei* hailing from a particular region of the country with foreign strains and present a comparative analysis to identify essential genes involved in their pathogenicity, adaptability, and drug resistance mechanisms.

Declaration of interest:

The authors declare no conflict of interest.

Author Contribution:

Conceived and designed the experiments: JAH, MSAJ, AT, CRA.

Performed the experiments: MSAJ, AT.

Analyzed the data: MSAJ, SF, AB, LB.

Contributed to reagents/materials/analysis tools: MSAJ, AT, AB, LB.

Wrote the paper: MSAJ, SF, AB.

Acknowledgements:

We thank Dr. Michael Norris, a former Postdoctoral Associate in Dr. A. Tuanyok's Laboratory, at the University of Florida for his technical assistance. We would also like to thank Dr. Mili Rani Saha, Dr. Tanzila Rahman and Md. Rokib Hasan, Department of Microbiology, BIRDEM Hospital for their assistance in detection, identification and preservation of the strains.

References:

1 Dance DA, Puthucheary SD. Epidemiology. In: Keethesan N, editor. Melioidosis: A century of observation and research. Amsterdam: Elsevier B.V.; 2012. p. 24.

2 Cheng AC, Currie BJ. Melioidosis: epidemiology, pathophysiology, and management. *Clinical microbiology reviews*. 2005; **18**(2): 383–416.

3 Dance DA. Melioidosis as an emerging global problem. *Actatropica*. 2000; **74**(2-3): 115–9.

4 Currie BJ, Dance DA, Cheng AC. The global distribution of Burkholderia pseudomallei and melioidosis: an update. *Transactions of the Royal Society of Tropical Medicine and Hygiene*. 2008; **102**: 1–4.

5 Govan BL. Molecular characterization and classification of Burkholderia pseudomallei. In: Keethesan N, editor. Melioidosis: A century of observation and research. Amsterdam: Elsevier B.V.; 2012: 60–67.

6 Webb JR, Win MM, Zin KN, Win KK, Wah TT, Ashley EA, et al. Myanmar Burkholderia pseudomallei strains are genetically diverse and originate from Asia with phylogenetic evidence of reintroductions from neighbouring countries. *Scientific reports*. 2020; **10**(1): 1–0.

7 Sarovich DS, Garin B, De Smet B, Kaestli M, Mayo M, Vandamme P, et al. Phylogenomic analysis reveals an Asian origin for African Burkholderia pseudomallei and further supports melioidosis endemicity in Africa. *MSphere*. 2016; **1**(2): e00089–15.

8 Nandi T, Tan P. The Burkholderia pseudomallei genome- an emerging model for microbial complexity and pathogen virulence. In: Keethesan N, editor. Melioidosis: A century of observation and research. Amsterdam: Elsevier B.V.; 2012: 68–81.

9 Tuanyok A, Auerbach RK, Brettin TS, Bruce DC, Munk AC, Detter JC, et al. A horizontal gene transfer event defines two distinct groups within Burkholderia pseudomallei that have dissimilar geographic distributions. *Journal of bacteriology*. 2007; **189**(24): 9044–9.

10 Baker A, Pearson T, Price EP, Dale J, Keim P, Hornstra H, et al. Molecular phylogeny of Burkholderia pseudomallei from a remote region of Papua New Guinea. *PLoS One*. 2011; **6**(3): e18343.

11 Pearson T, Giffard P, Beckstrom-Sternberg S, Auerbach R, Hornstra H, Tuanyok A, et al. Phylogeographic reconstruction of a bacterial species with high levels of lateral gene transfer. *BMC biology*. 2009; **7**(1): 1–4.

12 Barai L, Jilani MS, Haq JA. Melioidosis case reports and review of cases recorded among Bangladeshi population from 1988-2014. *Ibrahim medical college journal*. 2014; **8**(1): 25–31.

13 Barai L, Saha MR, Rahman T, Sukanya M, Jafrin J, Ferdous J, et al. Trends in laboratory detection of Burkholderia pseudomallei and their antibiotic susceptibility pattern from clinical samples over twenty-one years from 2001 to 2021 in a tertiary care hospital of Bangladesh. *Bangladesh journal of medical microbiology*. 2021; **15**(1): 8–14.

14 Maegraith BG, Leithead CS. Melioidosis: A Case-Report. *Lancet*. 1964; 862–3.

15 Jilani MS, Robayet JA, Mohiuddin M, Hasan MR, Ahsan CR, Haq JA. Burkholderia pseudomallei: its detection in soil and seroprevalence in Bangladesh. *PLoS neglected tropical diseases*. 2016; **10**(1): e0004301.

16 Dale J, Price EP, Hornstra H, Busch JD, Mayo M, Godoy D, et al. Epidemiological tracking and population assignment of the non-clonal bacterium, Burkholderia pseudomallei. *PLoS neglected tropical diseases*. 2011; **5**(12): e1381.

17 Currie BJ, Mayo M, Anstey NM, Donohoe P, Haase A, Kemp DJ. A cluster of melioidosis cases from an endemic region is clonal and is linked to the water supply using molecular typing of Burkholderia pseudomallei isolates. *The American journal of tropical medicine and hygiene*. 2001; **65**(3): 177–9.

18 Ashdown LR. An improved screening technique for isolation of Pseudomonas pseudomallei from clinical specimens. *Pathology*. 1979; **11**(2): 293–7.

19 Novak RT, Glass MB, Gee JE, Gal D, Mayo MJ, Currie BJ, et al. Development and evaluation of a real-time PCR assay targeting the type III secretion system of Burkholderia pseudomallei. *Journal of clinical microbiology*. 2006; **44(**1): 85–90.

20 Godoy D, Randle G, Simpson AJ, Aanensen DM, Pitt TL, Kinoshita R, et al. Multilocus sequence typing and evolutionary relationships among the causative agents of melioidosis and glanders, Burkholderia pseudomallei and Burkholderia mallei. *Journal of clinical microbiology*. 2003; **41**(5): 2068–79.

21 Jolley KA, Bray JE, Maiden MC. Open-access bacterial population genomics: BIGSdb software, the PubMLST. org website and their applications. *Wellcome open research*. 2018; **3**.

22 Zhou Z, Alikhan NF, Sergeant MJ, Luhmann N, Vaz C, Francisco AP, et al. GrapeTree: visualization of core genomic relationships among 100,000 bacterial pathogens. *Genome research*. 2018; **28**(9): 1395–404.

23 Argimón S, Abudahab K, Goater RJ, Fedosejev A, Bhai J, Glasner C, et al. Microreact: visualizing and sharing data for genomic epidemiology and phylogeography. *Microbial genomics*. 2016; **2**(11).

24 Saitou N, Nei M. The neighbor-joining method: a new method for reconstructing phylogenetic trees. *Molecular biology and evolution*. 1987; **4**(4): 406–25.

25 Letunic I, Bork P. Interactive tree of life (iTOL) v3: an online tool for the display and annotation of phylogenetic and other trees. *Nucleic acids research*. 2016; **44**(W1): W242–5.

26 Feil EJ, Li BC, Aanensen DM, Hanage WP, Spratt BG. eBURST: inferring patterns of evolutionary descent among clusters of related bacterial genotypes from multilocus sequence typing data. *Journal of bacteriology*. 2004; **186**(5): 1518–30.

27 Rajendra KC, O'Toole RF. Understanding the Biology of Non-typeable Haemophilus influenzae in Chronic Obstructive Pulmonary Disease Through the Lens of Genomics. In: Dua K, Lobenberg R, Luzo ACM, Shukla S, Satija S, editors. Targeting Cellular Signaling Pathways in Lung Diseases. Singapore: Springer; 2021. pp. 471–484.

28 Chewapreecha C, Holden MT, Vehkala M, Valimaki N, Yang Z, Harris SR, et al. Global and regional dissemination and evolution of Burkholderia pseudomallei. *Nature microbiology*. 2017; **2**(4): 1–8.

29 Cheng AC, Hanna JN, Norton R, Hills SL, Davis J, Krause VL, et al. Melioidosis in northern Australia, 2001-02. *Communicable diseases intelligence*. 2003; **27**(2): 4–17.

30 White NJ. Melioidosis. *The Lancet*. 2003; **361**(9370): 1715–22.

31 Zhu X, He L, Kuang H, Li H, Li S, Chen H, et al. Correlation between climatic factors and incidence of melioidosis in Sanya, Hainan. *Disease surveillance*. 2020; **35**(2): 156–61.

32 Zueter AR, Rahman ZA, Abumarzouq M, Harun A. Multi locus sequence typing of clinical *Burkholderia pseudomallei* isolates from peninsular Malaysia and their associations disease outcomes. *BMC infectious diseases*. 2018; **18**: 5.

33 Balaji V, Perumalla S, Perumal R, Inbanathan FY, Rajamani Sekar SK, Paul MM, et al. Multi locus sequence typing of Burkholderia pseudomallei isolates from India unveils molecular diversity and confers regional association in Southeast Asia. *PLoS neglected tropical diseases*. 2018; **12**(6): e0006558.

34 Fang Y, Zhu P, Li Q, Chen H, Li Y, Ren C, et al. Multilocus sequence typing of 102 Burkholderia pseudomallei strains isolated from China. *Epidemiology & Infection*. 2016; **144**(9): 1917–23.

35 Struelens MJ, Mondol G, Bennish M, Dance DA. Melioidosis in Bangladesh: a case report. *Transactions of the Royal Society of Tropical Medicine and Hygiene*. 1988; **82**(5): 777–8.

36 Limmathurotsakul D, Golding N, Dance DA, Messina JP, Pigott DM, Moyes CL, et al. Predicted global distribution of Burkholderia pseudomallei and burden of melioidosis. *Nature microbiology*. 2016; **1**(1): 1–5.

37 Idris A, Rachmat RF, Ali SM. Melioidosis: a case of sheep to human transmission. *Jurnal Veterinar Malaysia*. 1998; **10**(2): 77–9.

Figure 1

Click here to access/download;Figure;figure 1.jpg

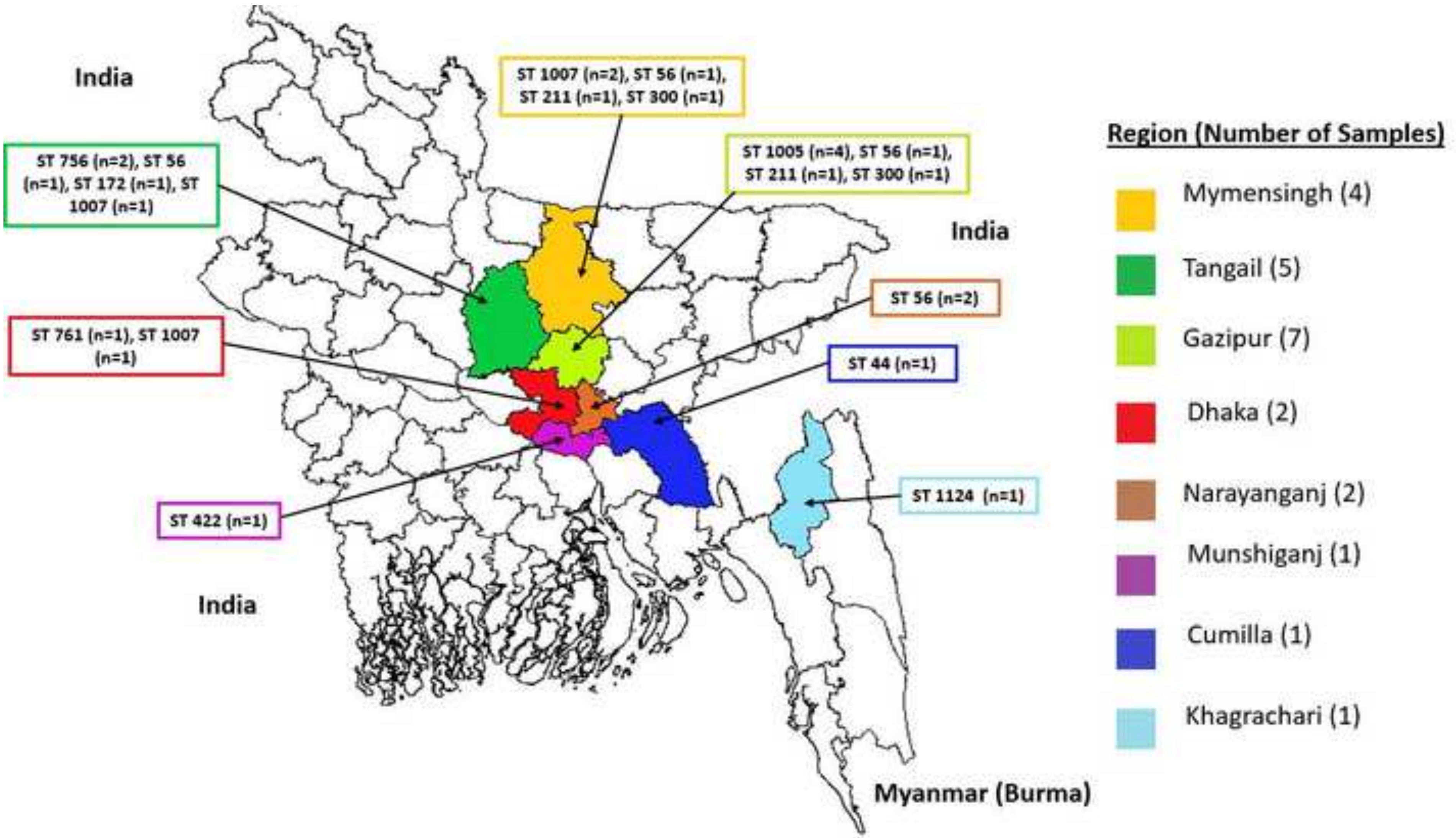

Figure 2

Click here to access/download;Figure;figure 2.jpg

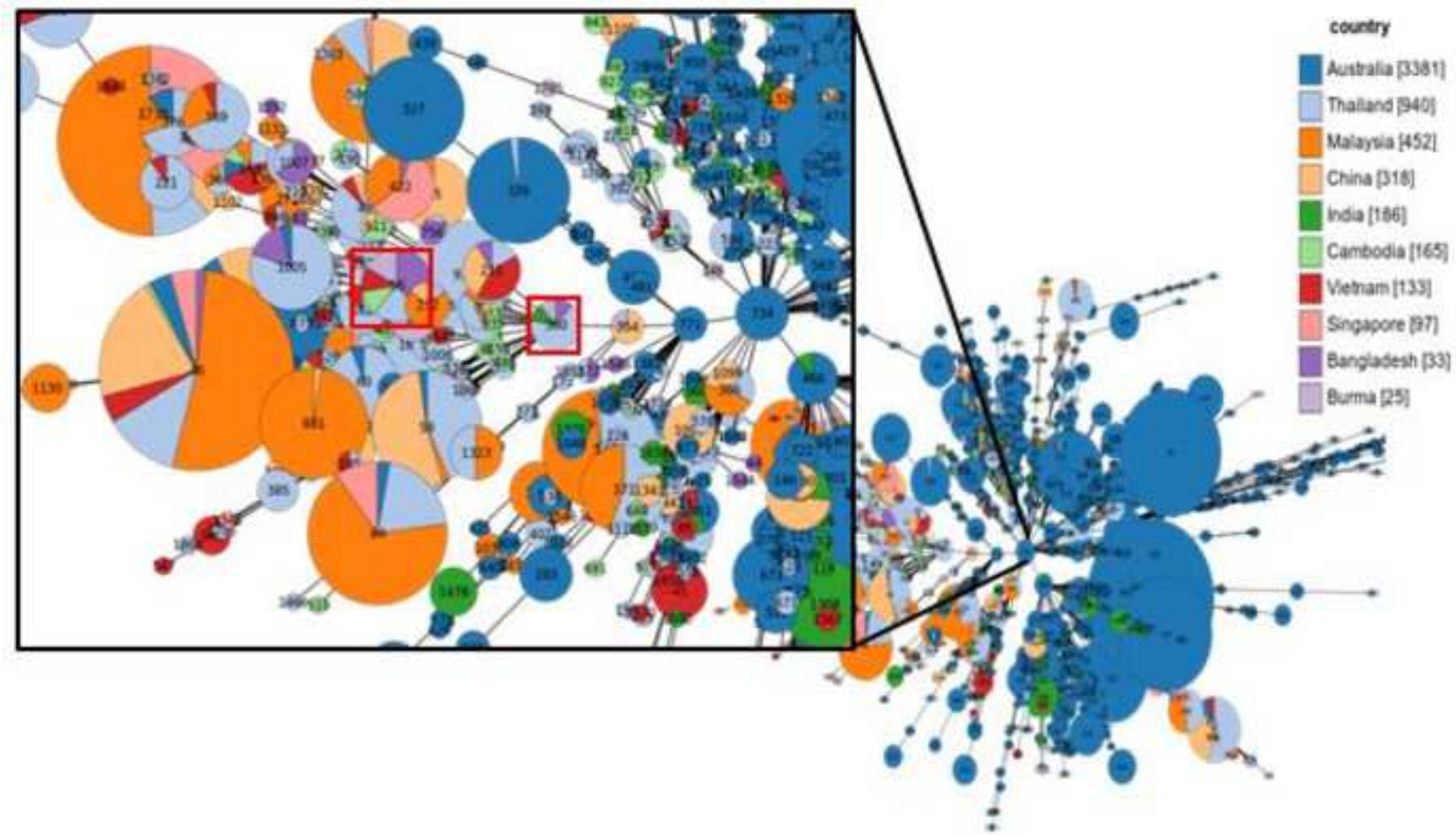

Figure 3

Click here to access/download;Figure;figure 3.jpg

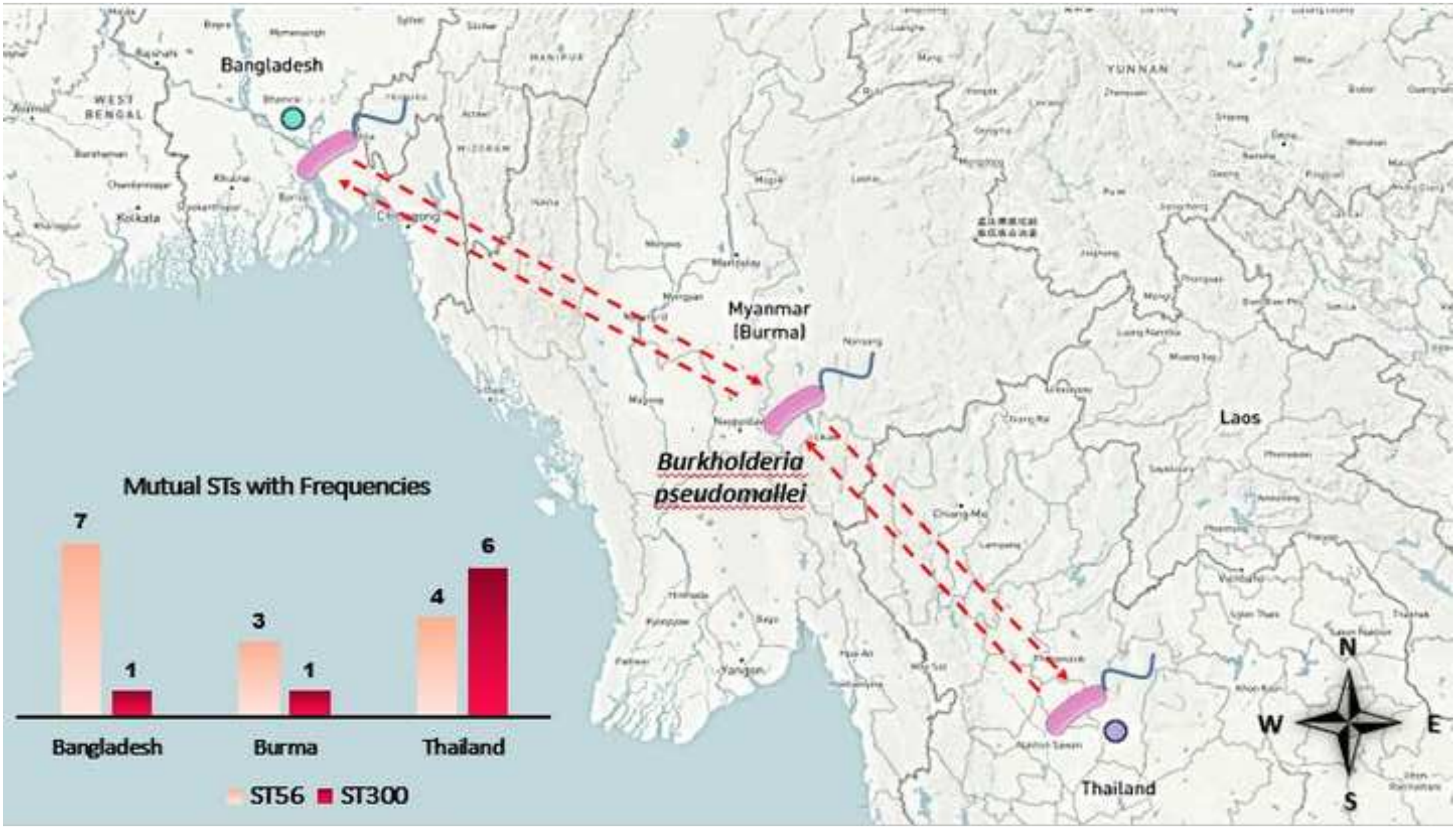

Figure 4

Click here to access/download;Figure;figure 4.jpg

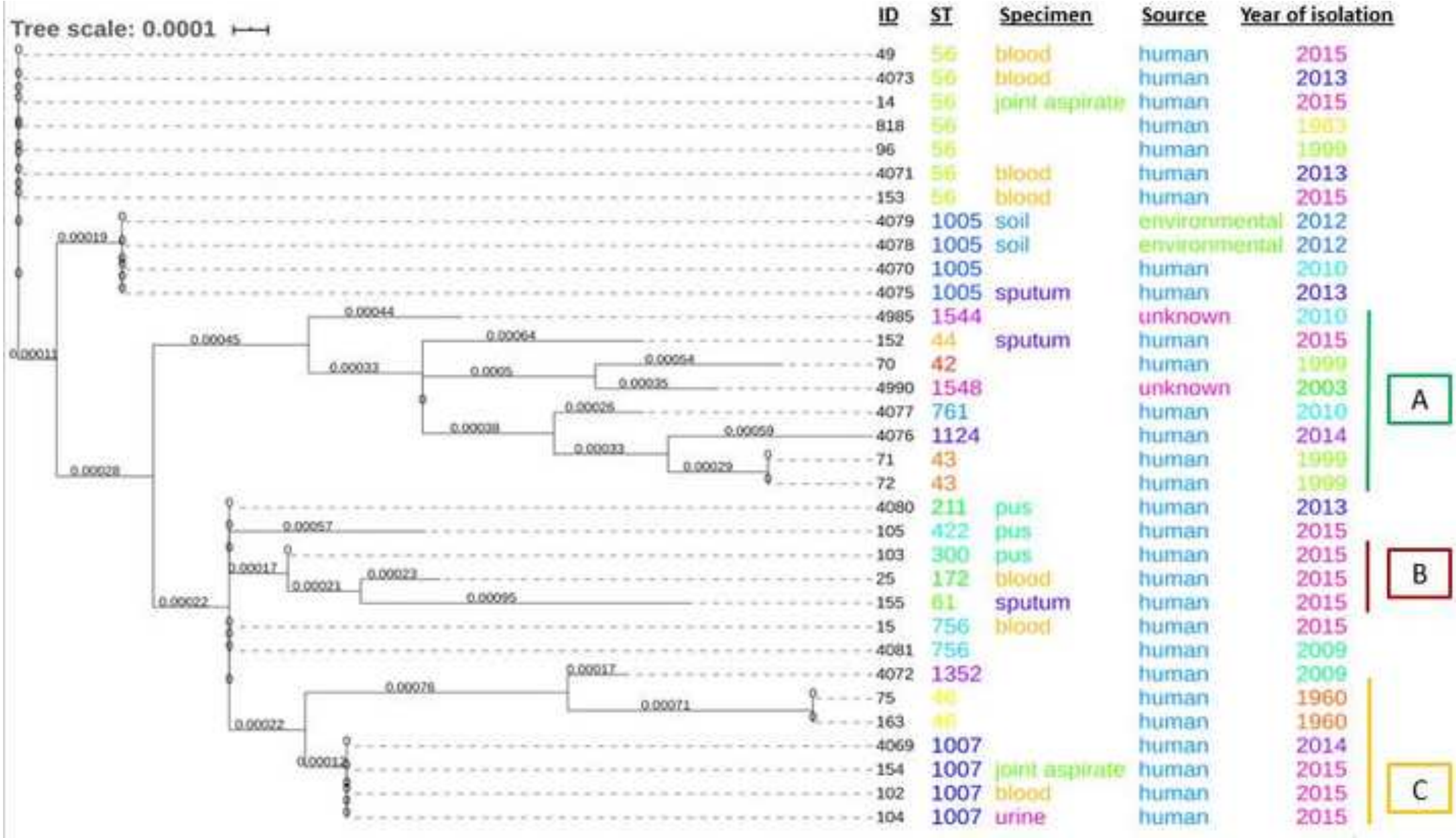

Supplementary Materials

Click here to access/download
Supporting Information
Supplementary Materials.pdf
