# Supplementary material for: Phylogeographic characterization of Burkholderia pseudomallei isolated from Bangladesh
Source: PLoS Negl Trop Dis. 2023 Dec 7;17(12):e0011823. doi: 10.1371/journal.pntd.0011823 (PMC10729972; doi:10.1371/journal.pntd.0011823)
Supplement: Attachment — Submitted filename: PNTD review RW.docx [file pntd.0011823.s007.docx]

PNTD-D-22-00909 entitled “Phylogeographic characterization of *Burkholderia pseudomallei* isolated from Bangladesh” This study typed 25 *B. pseudomallei* isolates, of which 22 are clinical, 2 are soil isolates and 1 reference isolate from Malaysia, using multilocus sequence typing (MLST) and construct a phylogenetic tree.

Major comments:

1. The number of isolates used in this study is very limited. All the clinical isolates were obtained from DM patients in BIRDEM General Hospital from 2009 to 2015. Only 2 isolates were obtained from the environment of the same place and the same year.

Most of the patients were reported to come from Gazipur and Tangail and 2 soil isolates also from Gazipur. Moreover, the sources of the isolates may not fit the title “phylogeographic characterization” even though the patients who attended that hospital came from different regions but near by the hospital. This also created a bias of ST types obtained in this study.

2. The MLST typing from 7 housekeeping genes sequences is a very useful molecular tool to subtype the organism and compared them with isolates from around the world. However, interpretation and conclusion of isolates with the same ST should be careful and not to be over claim.

For example, “ST 1005 was detected in both soil and clinical samples of Gazipur which indicates the soil to human transmission of this pathogen*”* is not appropriate.

Lines 156: “However, according to PubMLST database we found, for the first time, two isolates with ST 1005 from clinical samples of humans residing in the same district” doesn’t make any sense.

Lines 241-243: “Only, ST 1124 and ST 300 were common between India and Bangladesh. Therefore, most plausibly, the major introduction of *B. pseudomallei* took place via Southeast Asian regions in Bangladesh.” This may be over claimed.

3. Discussion needs more attention as the authors do not carefully interpret their results in scientific way of thought.

for example, lines 213-218: In Thailand, where YLF is dominant, mortality rates for the primary disease are higher (50%) than the endemic regions of Australia, where mortality rate is around 15 to 20% [29,30]. In an earlier study conducted in Bangladesh, sero-positivity rate among healthy individual was found to be 22·6%-30·8% [15]. If reactivation occurs in these cases due to an immune-compromised state, the YLF dominance may play a vital role in elevating the mortality rates.”

The mortality rate depends on several factors in each country that infecting with the bacterium that carrying YLF genotype cannot indicate the outcome. Moreover, the idea if the reactivation occurred and extrapolate it to link with mortality rate is not suitable.

About ST 56 that commonly found in Bangladesh, the author mention in line 250 that ST 56 was also isolated from a patient in 1983. This requires a reference. Moreover, (lines 253-254) the presence of this ST type in the countries nearby is not enough to indicate that *B. pseudomallei* may spread from these countries to Bangladesh. Lines 256-257 concluded that ST 56 is virulence as all isolates with this ST type were isolated from patients’ blood and 2 out of 5 died. These interpretation and conclusion are not scientifically sound.

Minor comments

1. Figure 2 and 3 needs more details for the interpretation and further clarify.
2. The information about each isolate were listed together with phylogenic tree in figure 4 that quite difficult to read.
