# Supplementary material for: Phylogeographic characterization of Burkholderia pseudomallei isolated from Bangladesh
Source: PLoS Negl Trop Dis. 2023 Dec 7;17(12):e0011823. doi: 10.1371/journal.pntd.0011823 (PMC10729972; doi:10.1371/journal.pntd.0011823)
Supplement: Attachment — Submitted filename: Letter adressing the reviewers comments 2nd version.docx [file pntd.0011823.s008.docx]

Manuscript Number: PNTD-D-22-00909

Full Title: Phylogeographic characterization of *Burkholderia pseudomallei* isolated from

Bangladesh

Short Title: Molecular typing of *Burkholderia pseudomallei* in Bangladesh

Article Type: Research Article

The Editor,

Plos NTD

We thank the reviewers for their valuable opinions regarding the article. We have tried to elaborately address the comments and queries made by the reviewers. We have separately addressed the specific comments, the major and minor comments as well as comments made by reviewer-4 individually. In addition, since the article is part of a project that we are working on, further research demonstrated that there was discrepancy in two of our isolates (CS60 and CS4987), which have been removed from the article. In doing so, the sequence types assigned to these two isolates ST 300 and ST44 have also been removed and the data re-analysed and incorporated, for which some major changes have been made, that we are including in this letter. The corrected manuscript have been attached, one with track change mode and another clean version as per requirement. In addition, we are also attaching the figures again that have been corrected as per the new data analyses (TIFF file). We hope we could successfully address all the queries made by the reviewers and we are eagerly waiting for a positive response.

Thanking you

Prof. Md. Shariful Alam Jilani (Corresponding author)

Professor and Head

Department of Microbiology

Ibrahim Medical College

Dhaka, Bangladesh

**Major corrections made by the authors:**

This article is a part of a melioidosis project that we are conducting in Bangladesh. On further progression of our research work, we found discrepancy with two of our clinical isolates CS60 and CS4987 (ST 300 and ST 44 respectively), for which all data relevant to these two isolates have been omitted from the article. Furthermore, since two sequence types have been removed we re-analysed all the data again following which major changes were made with our GrapeTree analysis, BURST analyses as well as phylogenetic tree that we have tried to incorporate in details in the methodology, result and discussion section.

**Abstract**

**Method: “**In this study, 24 *B. pseudomallei* isolates, of which 22 clinical and two soil isolates…” has been corrected to “In this study, 22 *B. pseudomallei* isolates, of which 20 clinical and two soil isolates..” as CS60 and CS4987 isolates have been removed from the study.

Findings: “A total of 13 different Sequence Type (ST) that includes five novel STs.. has been corrected to “A total of 12 different Sequence Type (ST) that includes four novel STs…”

**Introduction:**

Line 64-65: “These factors have led to distinct Asian and Australasian *B. pseudomallei* populations that have regionally evolved over hundreds or thousands of years.” has been deleted from the manuscript.

**Methodology:**

Line 98-99: “a total of 25 *B. pseudomallei* isolates that included 22 clinical,” has been changed to “The study utilized a total of 23 *B. pseudomallei* isolates that included 20 clinical, two soil isolates..” (line-97 in new manuscript) as CS60 and CS4987 isolates have been removed from the study.

**Result:**

Line 138: “Most of the patients came from Gazipur and Tangail..” has been changed to

Out of 22, 6 patients came from Gazipur while 5 patients were from Tangail.” (lines- 135-136 in new manuscript)

Line 143: “Real-time PCR assay, targeting TTS1, confirmed that all 22 clinical..” has been changed to “Real-time PCR assay, targeting TTS1, confirmed that all 20 clinical..” (line-140 in new manuscript)

Line 149: “MLST unveiled a total of 13 ST of which five ST (ST- 756, 1352, 761, 44 and 61) were novel types…” has been changed to “MLST unveiled a total of 12 ST of which five ST (ST- 756, 1352, 761 and 61) were novel types…” (line-146 in new manuscript)

Line 165-170: “Most of the isolates of this study are connected genetically to ST 300. ST

300 has been found in Thailand, India and Myanmar. Relatives of ST 300 such as ST 56,

1005 and 1007 are predominant in Bangladesh. These ST have also been reported in

Myanmar, Thailand, Cambodia, Vietnam and Australia. However, none of these ST were

found in the neighbouring country India.”- the paragraph has been omitted since the

GrapeTree has been plotted again omitting ST 300 and ST 44. The new findings has been

added in the lines- “According to the Grape Tree of PubMLST, two distinctive clusters were

observed. In one cluster Australian strains such as ST 553, ST 131 were present, while in the

other cluster, all strains were connected to Australian/ Asian strain ST 562. This ST 562

generated cluster is less predominant with Australian isolates and seems more diverse.” (lines-

168-171 in new manuscript)

Line 173: “ST 300” has been deleted.

Line 176-177: “Most of the sequence types (ST) of Bangladesh are connected to ST300 (Labeled in red box). Relatives of ST-300 such as ST-56, 1005 and 1007 are mostly prevalent in Bangladesh.”- has been omitted from the caption of Figure 2 since the figure has been re-drawn. (line- 176-177 in new manuscript)

Line 186: “also” has been omitted. (line-179 in new manuscript)

Line 187: “Five novel genotypes (ST-1352, 44,756, 61 and 761) were found in this study” has been corrected to- “Four novel genotypes (ST-1352,756, 61 and 761) were found in this study.” (line-180 in new manuscript)

Line 187: “ST” has been re-written as “sequence types” (lines-180-181 in new manuscript)

Line 188: “ST 44” has been removed (line-181 in new manuscript).

Line 187-188: The line “Among the ST of Bangladesh, ST-44 and 761 clustered in clade A” has been omitted and written as “Among the sequence types of Bangladesh, ST- 761 is clustered along with ST 1124 and and ST 1548.” (lines-180-181 in new manuscript)

Lines 189-190: “ST 756 did not cluster in any clades, however, demonstrated close relation with clade B”- has been re-written as “Sequence Type 756 did not cluster in any clades.” (line-182-183 in new manuscript)

Line 191: “44” has been deleted. (line-184 in new manuscript)

Line 193: “ST 44 is a DLV of ST 314, while..” has been deleted.

Line 198: “Here, some of the isolates have clustered together and generated clades A, B and C.” -has been deleted from caption of figure-4 (figure-3 in new manuscript). (line-191 in new manuscript)

**Discussion**

Line 215: “.” has been replaced by “;” (line-206 in new manuscript)

Line 219: “A total of 13 different sequence types (ST) was found by MLST” has been omitted.

Line 220: “ST” has been written as “sequence types” (line-210 in new manuscript)

Line 221: “(Figure 4)” has been replaced by “(Figure 3)” (line-211 in new manuscript)

Line 222: “ST” has been replaced by “Sequence Types” (line-212 in new manuscript)

Line 227: “According to PubMLST, at least five ST (ST-1352, 44, 756, 761 and 61)..” has been replaced by “According to PubMLST, at least four ST (ST-1352, 756, 761 and 61)..” (line-218 in new manuscript)

Lines 230-232: “Most of these novel ST were single locus variant and double locus variant of strains from Thailand, which indicate the clonal expansion of their ancestors in this region were due to endemicity.” has been omitted.

Line 236: “Apparently, similar pattern was also observed after MLST profiling in our isolates.” has been omitted.

Lines 237-239: “All of the isolates are genetically related with ST 300 and ST 354, which have been found in Southeast Asian regions (Figure 3).” has been omitted. Instead the new analyses revealed a different finding which has been incorporated as- “The Grape Tree clusters suggest that Australian strains such as ST 553, ST 131 present are distinctive from Australian/ Asian strain ST 562 which is related with the strains found in Bangladesh (Figure 2). Most plausibly, ST 562 went under different selection pressure and their variants were introduced in Bangladesh via tropical south-eastern pasrt of Bangladesh (Myanmar and China).” (Lines- 225-229 in corrected manuscript).

Line 239: “ST 300” has been deleted and “ST 56” has been re-written as “Sequence Type 56” and the line corrected as “GrapeTree also reveals Sequence Type 56 are isolated …” (Line- 229 in corrected manuscript).

Line 240: “In addition, other ST like ST 172, ST 211, ST 1005 and ST 1007” has been replaced by “In addition, other sequence types like ST 172, ST 211 and ST 422…” in accordance with new GrapeTree analysis. (Lines- 230-231 in corrected manuscript).

Line 242: “ST 300” has been deleted. (Line- 232 in corrected manuscript).

Table 1:ST 300 and ST 44 has been removed.

Supplementary table 1: CS60 and CS4987 has been removed.

Figure 3 has been deleted. Figures 1, 2 and 4 (new Figure 3) have been corrected and uploaded as tiff folders.

**Responses to the specific comments with regards to the text of the manuscript are as follows:**

| S/No. | Section | Lines | Remarks |  |
| --- | --- | --- | --- | --- |
| 1. | Title |  |  |  |
| 2. | Abstract |  | Significance: ST 1005 was detected in both soil and clinical samples of Gazipur which indicates the soil to human transmission of this pathogen.  Comments: The organism is already known soil dwelling pathogen | “which indicates the soil to human transmission of this pathogen” has been omitted from the abstract. |
| 3. |  |  | This type of strains may have originated from Vietnam------- Comments: There are no enough evidences to suggest strain spread from Vietnam to Bangladesh as far as this work is concerned. | “This type of strains may have originated from Vietnam and spread via Myanmar to Bangladesh but further identification of isolates carrying ST 56 are required to confirm this hypothesis.”- has been omitted from the abstract. |
| 4. | Introduction | 37-38 | Thailand and Singapore, where extensive surveillance had been undertaken.  Comments: The authors need to cite some references of those “extensive surveillance” mentioned. | The references has been cited: Introduction line 35 in corrected version.  Reference section- lines 282-288 in corrected version. |
| 5. |  | 99 | ----- one reference strain (USM) obtained from Universiti Sans Malaysia.  Comments: Authors need to give the identity of the isolate from USM for reference purpose e.g USM??? Or No.. | The reference strain is known as the USM strain. For further clarification we have mentioned “USM strain” within brackets (line-98 in corrected version). |
| 6. | Methodology | 101 | ------- BIRDEM General Hospital from 2009 to 2015 and two soil strains isolated from-------  Comments: “BIRDEM” if this is an abbreviation it should be written in full the abbreviation in apprentices. | The full form of BIRDEM has been included as Bangladesh Institute of Research and Rehabilitation in Diabetes, Endocrine and Metabolic Disorders (lines- 100-101 in corrected version). |
| 7. |  | 118 | MLST was performed according to the methods of Godoy et  al. ----------  Comments: the citation should follow the PNTD referencing format. | “according to the methods of Godoy et al.”- the lines has been omitted. The reference is cited [25..] in line-118 in corrected version. |
| 8. | Result | 138 | Most of the patients came from Gazipur and Tangail.  Comments: Since the work is solely on Phylogeography, the authors need to give location-specific prevalence of the 22 isolates. | The line has been re-written as “Out of 20, 6 patients came from Gazipur while 5 patients were from Tangail.”  Lines- 134-135 in corrected version. |
| 9. |  | 166-  167 | ST 300 has been found in Thailand, India and Myanmar.  Comments: ST should be in full at the beginning of sentence. | “ST 300 has been found in Thailand, India and Myanmar”- the line has been omitted from the manuscript as we removed ST 300 due to discrepancy found with the particular isolate. |
| 10. |  | 172 | Supplementary Figure 1  Comments: Why not Figure 1 instead of Supplementary  Figure 1.Since subsequent figures in 174 is referred to Figure 2 | Supplementary Figure-1 is provided with the Supplementary file. It is not included in the figure submissions. Supplementary figure-1 shows the close relationship among the isolates from Bangladesh, Thailand and Myanmar that are differentiated in three different colors. On the contrary, Figure-2 is separately submitted as Figure file. |
| 11. |  | 174 | ST 56 is predominant in both Bangladesh and Myanmar  Comments: ST should be in full at the beginning of sentence. | Corrected as ‘Sequence Type” in lines 173-174 in corrected version. |
| 12. |  | 188 | ST 1352 clustered with the clade of-------- Comments: Same as 174 | Corrected as ‘Sequence Type” in line 180-181 in corrected version. |
| 13. |  | 189 | ST756 did not cluster in any clades, however, demonstrated close relation with-------- Comments: Same as 174 | Corrected as ‘Sequence Type” in line 181 in corrected version. |
| 14. |  | 192-  194 | Supplementary Figure 2 and Supplementary Figure 1, including Supplementary Figure 1 were not among the figures presented in the manuscript. Since they are part of the results presented they should be included. | The resolutions of the supplementary figures are blurry even following adjustments in 300 pixels/inch for which they were not included in the main manuscript rather incorporated as pdf files. However, for convenience we are attaching all figures as a ppt folder. |
| 15. | Discussions | 219-222 | Comments: This section is a repetition of the results presentation in discussion. | “A total of 13 different sequence types (ST) was found by MLST.”-this line has been omitted as it was a repetition of result section. |
| 16. |  | 222 | ST 1005, ST 56, ST 1007 and ST 211 have been detected not only in Bangladesh-------------  Comments: Same as 174 | Corrected as ‘Sequence Types” in line 211 in corrected version. |
| 17. |  | 225 | ST 56 which was present in five clinical 225 isolates is------ Comments: Same as 174 | Corrected as ‘Sequence Type” in line 214 in corrected version. |
| 18. |  | 264-  268 | An important feature of melioidosis is the spatial clustering of clinical incidence which is linked to the environmental prevalence of the organism-------  Comments: This section is not relevant to this work as no environmental samples were collected to correlate with soil types | The line, “An important feature of melioidosis is the spatial clustering of clinical incidence which is linked to the environmental prevalence of the organism.”- has been omitted. However, the following lines were kept as it is as we wanted to imply that *B. pseudomallei* isolated from Gazipur were from modified soil (anthrosol) rather than clay-rich acrisol type of soil (lines 250-251 in corrected version). |
| 19. |  | 275-  277 | B. pseudomallei possessing ST 46 was recovered in 1960 from a crewman travelling through----------  Comments: This section is redundant as this work did not recovered ST46. | *“B. pseudomallei* possessing ST 46 was recovered in 1960 from a crewman travelling through…”- the paragraph has been omitted. |
| 20. |  | 283-  286 | It is likely that human and/or animal movements between these areas--------  Comments: This section is irrelevant and redundant as no animal isolates are involved in this study nor animal movements | *“B. pseudomallei* possessing ST 46 was recovered in 1960 from a crewman travelling through…”- the paragraph has been omitted. |

**Responses to Major Comments:**

1. The number of isolates used in this study is very limited. All the clinical isolates were obtained from DM patients in BIRDEM General Hospital from 2009 to 2015. Only 2 isolates were obtained from the environment of the same place and the same year.

Most of the patients were reported to come from Gazipur and Tangail and 2 soil isolates also from Gazipur. Moreover, the sources of the isolates may not fit the title “phylogeographic characterization” even though the patients who attended that hospital came from different regions but near by the hospital. This also created a bias of ST types obtained in this study.

**Response:** BIRDEM General Hospital is a tertiary care reference hospital in Bangladesh for diabetic patients (BIRDEM is an abbreviation for Bangladesh Institute of Research and Rehabilitation in Diabetes, Endocrine and Metabolic Disorders, included in lines- 100-101). As we know, Diabetes Mellitus is a major risk factor for melioidosis. In this study, the patients infected with *B. pseudomallei* that were isolated in Bangladesh in a recorded form belonged to BIRDEM only, as any other district level hospitals did not notify any case of melioidosis till the study period. Furthermore, the diabetic patients that arrive in BIRDEM are actually from all over the country and not from nearby regions only.

Moreover, soil samples were collected from 4 districts of Bangladesh systematically, of which only 2 isolates of *B. pseudomallei* were identified. This work is mentioned in details in a previous article by the same author (Jilani et al., 2015). Under these circumstances, we were trying to emphasize on the word “phylogeography” as it carries a geographic aspect of the isolates as well in addition to molecular typing.

2. The MLST typing from 7 housekeeping genes sequences is a very useful molecular tool to subtype the organism and compared them with isolates from around the world. However, interpretation and conclusion of isolates with the same ST should be careful and not to be over claim.

For example, “ST 1005 was detected in both soil and clinical samples of Gazipur which indicates the soil to human transmission of this pathogen*”* is not appropriate.

Lines 156: “However, according to PubMLST database we found, for the first time, two isolates with ST 1005 from clinical samples of humans residing in the same district” doesn’t make any sense.

Lines 241-243: “Only, ST 1124 and ST 300 were common between India and Bangladesh. Therefore, most plausibly, the major introduction of *B. pseudomallei* took place via Southeast Asian regions in Bangladesh.” This may be over claimed.

**Response:**

Abstract- significance section: “which indicates the soil to human transmission of this pathogen” has been omitted from the abstract.

Lines 156: the line has been changed to “According to PubMLST database, ST 1005 was previously isolated from soil samples of different regions such as environmental samples from Thailand and Australia. Interestingly, ST 1005 has not been isolated from clinical samples previously from any patients of any country. In Bangladesh, we have isolated two ST 1005 *B. pseudomallei* genotypes for the first time, from sputum and urine samples respectively. Whole genome sequencing study can provide further information about any possible linkage between the soil and clinical ST 1005 isolates.”- lines 149-154 in corrected version.

Lines 242-243: “Therefore, most plausibly, the major introduction of *B. pseudomallei* took place via Southeast Asian regions in Bangladesh.”- has been omitted.

3. Discussion needs more attention as the authors do not carefully interpret their results in scientific way of thought.

for example, lines 213-218: In Thailand, where YLF is dominant, mortality rates for the primary disease are higher (50%) than the endemic regions of Australia, where mortality rate is around 15 to 20% [29,30]. In an earlier study conducted in Bangladesh, sero-positivity rate among healthy individual was found to be 22·6%-30·8% [15]. If reactivation occurs in these cases due to an immune-compromised state, the YLF dominance may play a vital role in elevating the mortality rates.”

**Response:** This was just an observation, but since it appears an over claim, we have changed the sentence as follows: “In Thailand, where YLF is dominant, mortality rates for the primary disease are higher (50%) than the endemic regions of Australia, where mortality rate is around 15 to 20% [33,34], although high mortality rates are attributable to several confounding factors. Since the YLF gene is also dominant among the *B. pseudomallei* isolated in Bangladesh, further genomic studies may reveal whether virulence or disease severity is associated with the YLF gene that may lead to increase mortality rate.”(lines 203-208 in corrected version).

4. About ST 56 that commonly found in Bangladesh, the author mention in line 250 that ST 56 was also isolated from a patient in 1983. This requires a reference. Moreover, (lines 253-254) the presence of this ST type in the countries nearby is not enough to indicate that *B. pseudomallei* may spread from these countries to Bangladesh. Lines 256-257 concluded that ST 56 is virulence as all isolates with this ST type were isolated from patients’ blood and 2 out of 5 died. These interpretation and conclusion are not scientifically sound.

**Response:** About the ST 56 isolated from a patient in 1983, the data has been taken from PubMLST database. However, no related article was found for which a specific article’s reference could not be incorporated in the manuscript.

**“**This type of strains may have originated from Vietnam and spread via Myanmar to Bangladesh but further identification of isolates carrying ST 56 are required to confirm this hypothesis [6].”- the line has been omitted from the manuscript.

We have changed the line with ST 56 to “The five ST 56 that were isolated in Bangladesh were from patients’ blood”- lines 242-243 in corrected version, so that it does not appear non-scientific. However, we mentioned that there may be a linkage of virulence associated with ST 56 but that requires WGS study for confirmation mentioned in lines 246-249 in corrected version.

**Minor comments:**

We have removed figure 3 from our manuscript. However, about Figure-2 and the phylogenetic tree, we have changed it to TIFF instead of JPEG, keeping the resolution as 300 pixels/inch as per requirement of PLOS NTDs standards. The figures may still appear a little hazy when we try to zoom in and observe the data for which we are attaching the ppt file as well for your convenience.

**Responses to Reviewer 4:**

Abstract- first line of significance section-“which indicates the soil to human transmission of this pathogen” has been omitted from the abstract.

Abstract- third line of significance section- and discussion line-279, “strain” has been changed to “genotype.”

Introduction lines 38-39: “a disease of rural poor and usually affects the people in the area that are poorly supplied with the diagnostic capability to make the diagnosis, so the…” has been changed to “…a disease of rural poor that usually affects people in areas that are poorly supplied with the diagnostic capability to make a diagnosis, the…”

Introduction lines 44-47: The citations of 5 and 8 (now 7 and 10) has been changed to the original articles as follows:

Cheng AC, Godoy D, Mayo M, Gal D, Spratt BG, Currie BJ. Isolates of Burkholderia pseudomallei from northern Australia are distinct by multilocus sequence typing, but strains type do not correlate with clinical presentation. *Journal of Clinical Microbiology*. 2003; **42**: 5477-5483. (Reference lines 298-301)

Kim HS, Schell MA, Yu Y, Ulrich RL, Sarria SH, Nierman WC, et al. Bacterial genome adaptation to niches: divergence of the potential virulence genes in three Burkholderia species of different survival strategies. *BMC genomics*. 2005; **6**(1):1-3. (Reference lines 309-311).

Introduction lines 47-49: the lines has been changed from “From Asia *B. pseudomallei* eventually migrated to Madagascar and Africa and then through slave trading from West African Countries to America….” to “From Asia *B. pseudomallei* appears to have spread to Africa and from there to the Americas, possibly via slave trading from West Africa.” The citations has been corrected as [8,9].

Line 52: “among” has been changed to “within” (line 50 in corrected manuscript)

Line 54: “from Asia” has been changed to “from Asia and elsewhere” (line 52 in corrected manuscript)

Line 56: “associated” has been changed to “more associated” (line 54 in corrected manuscript)

Line 57: “clusters” has been changed to “cluster” (line 55 in corrected manuscript)

Lines 55-58: the reference has been changed as per suggestion, [11]. (line 56 in corrected manuscript)

Line 58: “This” has been changed to ‘The” (line 56 in corrected manuscript)

Lines 59-61: the citation for this lines has been added as [13] (lines 56-59 in corrected manuscript). In the reference section as: Hall CM, Jaramillo S, Jimenez R, Stone NE, Centner H, Busch JD, et al. Burkholderia pseudomallei, the causative agent of melioidosis, is rare but ecologically established and widely dispersed in the environment in Puerto Rico. *PLoS neglected tropical diseases*. 2019;**13**(9): e0007727 (lines 319-322).

Line 61: “via respiratory route” has been added. (lines 59-60 in corrected manuscript)

Line 62: “human-to-human transmission being exceptionally rare” has been supported by citation [14] (line 60 in corrected manuscript); reference section: Aziz A, Currie BJ, Mayo M, Sarovich DS, Price EP. Comparative genomics confirms a rare melioidosis human-to-human transmission event and reveals incorrect phylogenomic reconstruction due to polyclonality. *Microbial genomics*. 2020; **6**(2) (lines 323-325).

Line 63: “Australia, have..” has been changed to “Australia, may have..” (line 62 in corrected manuscript)

Line 66: the word “recent” has been deleted.

Lines 68-70: the lines “Till 2021, a total of 86 culture confirmed *B. pseudomallei* has been isolated from 68 melioidosis cases [13]. Although, the first case of melioidosis from Bangladesh is believed to be identified…” has been changed to “Until 2021, a total of 86 *B. pseudomallei* isolates have been obtained from 68 melioidosis cases [17]. Although, the first confirmed case of melioidosis from Bangladesh is believed to be identified…”(lines 65-67 in corrected manuscript)

Lines 74: In response to reviewer’s comment, the meaning of definite country for melioidosis has been explained and added in the manuscript with citation. “In 2013, melioidosis endemic countries were categorized into “definite” and “probable” on the basis of the presence of the causative organism in human as well as environment [20]. *B. pseudomallei* was isolated for the first time in Indian Subcontinent from soil samples from Gazipur district of Bangladesh. Since then, Bangladesh is considered as a definitive country for melioidosis due to the presence of *B. pseudomallei* in both clinical and soil samples [19].” (lines 69-74 in corrected manuscript)

Lines 75-79: the lines “…to melioidosis is unknown, due to a lack of extensive epidemiological surveillance program. For that reason, molecular analyses of *B. pseudomallei* isolates by multilocus sequence typing (MLST) may be used to examine the diversity of this organism from various geographic regions. This will assist to assess population structure of the organism as well as form phylogeographic characteristics that can be…” have been changed to “…to melioidosis are unknown, due to a lack of extensive epidemiological surveillance programs. Molecular analyses of *B. pseudomallei* isolates by multilocus sequence typing (MLST) can be used to examine the diversity of this organism from various geographic regions, which can assist with assessment of population structure of the organism as well as be…”(lines 75-78 in corrected manuscript)

Method: Why weren’t the isolated whole genome sequenced? The prospect of whole genome sequencing (WGS) is still quite new in Bangladesh. There is scarcity of NGS instrument, lack of trainings as well as bioinformaticians with an expertise in analysing the sequenced data. Commercially, WGS can be done in a few institutions but the study lacked the proper fund to conduct WGS of 22 isolates. However, the authors of this study are trying to set up a protocol and collaborate with scientists elsewhere to conduct the WGS of these isolates.

Methods line 118: the citation of Godoy is included as [25]. (line 118 in corrected manuscript)

Methods lines 127-128: “with the ST of Bangladesh” has been changed to “*B. pseudomallei* sequence types isolated from Bangladesh.” (line 125 in corrected manuscript)

Methods line 133: “genotypic behaviour” has been rephrased to “genotypic properties.” (line 131 in corrected manuscript)

Methods lines 137-138: the sentence that was a repetition of methodology (A total of 24 *B. pseudomallei* isolates were collected of which 22 were clinical, and two were environmental, from 2009 to 2015) has been deleted. The sentence has been modified to “Out of 20 patients from whom clinical samples were collected, 6 patients came from Gazipur while 5 patients were from Tangail.” (lines 134-135 in corrected manuscript)

Discussion lines 210-211: the sentence has been rephrased as “YLF gene cluster is horizontally acquired while BTFC gene cluster is ancestral to *B. pseudomallei* [11].”- with appropriate citation. (lines 199-200 in corrected manuscript)

Lines 213-215: “In Thailand, where YLF is dominant, mortality rates for the primary disease are higher (50%) than the endemic regions of Australia, where mortality rate is around 15 to 20%” (lines 203-205 in corrected manuscript)- this is just an observation made and we have included that mortality rates depend on several factors. We have included that further genomic studies may reveal whether virulence is associated with the YLF dominance. The sentence has been rephrased as “In Thailand, where YLF is dominant, mortality rates for the primary disease are higher (50%) than the endemic regions of Australia, where mortality rate is around 15 to 20% [29,30], although high mortality rates are attributable to several confounding factors. Since the YLF gene is also dominant among the *B. pseudomallei* isolated in Bangladesh, further genomic studies may reveal whether virulence or disease severity is associated with the YLF gene that may lead to increase mortality rate.” (lines 203-208 in corrected manuscript)

Discussion lines 215-218 and line 218: In response to reviewer’s comment- “Reactivation almost certainly would not occur in someone who has seroconverted”, the following lines have been deleted from the manuscript- “In an earlier study conducted in Bangladesh, sero-positivity rate among healthy individual was found to be 22·6%-30·8% [15]. If reactivation occurs in these cases due to an immune-compromised state, the YLF dominance may play a vital role in elevating the mortality rates.”

Discussion lines 241-243: “Therefore, most plausibly, the major introduction of *B. pseudomallei* took place via Southeast Asian regions in Bangladesh.”- has been omitted. We have made an observation in the following line but we mentioned that it can be only confirmed by further genomic studies. (lines 232-237 in corrected manuscript)

Discussion lines 259-261: We have changed the line with ST 56 to “The five ST 56 that were isolated in Bangladesh were from patients’ blood”- lines 242-243 in corrected manuscript. However, we mentioned that there may be a possibility of virulence linked with ST 56 but that requires WGS study for confirmation mentioned in lines 246-249.

Discussion lines 272-274: “In addition, this may also point out that naturally occurring groundwater functions as a vehicle for the dispersal of viable *B. pseudomallei* away from a primary environmental reservoir.”- the line has been deleted.

Figure 2 has been converted from jpeg to tiff format with 300 pixels/inch for ease of understanding.

Figure 3 has been deleted.

Figure 4 (Phylogenetic relationships of *B. pseudomallei* in Bangladesh. On the right side, the columns depict the ID of the strain in the PubMLST database, sequence type (ST), specimen, source of the isolate and year of isolation.) is the new figure 3 which has also been converted to tiff format and attached in the mail.
