# Supplementary material for: Phylogeographic characterization of Burkholderia pseudomallei isolated from Bangladesh
Source: PLoS Negl Trop Dis. 2023 Dec 7;17(12):e0011823. doi: 10.1371/journal.pntd.0011823 (PMC10729972; doi:10.1371/journal.pntd.0011823)
Supplement: Attachment — Submitted filename: Revised MS PNTD.docx [file pntd.0011823.s009.docx]

Revised MS PNTD-D-22-00909_R1

The revised manuscript title “Phylogeographic characterization of *Burkholderia pseudomallei* isolated from Bangladesh.” was mostly corrected and changed according to what reviewers’ suggestion.

There are a few more comments to be concerned.

As McRobb, E. et al, Applied and Environmental Microbiol, 2014 indicated when analyzed *B. pseudomallei* populations in Australia (779 clinical isolates, including 736 humans plus 43 animal isolates, and 174 environmental strains) using ST analysis that *B. pseudomallei* populations diversified as the sampling area increased. This observation contrasted with smaller sampling areas where a few STs predominated, suggesting that *B. pseudomallei* populations are ecologically established and not frequently dispersed. Therefore, the interpretation using a small number of isolates, even though sending from surrounding areas, in this study should mentioned for their limitation.

This manuscript mentioned about ST1005 that isolated from 2 clinical samples and this ST has not been isolated from clinical samples previously from any patients of any country. Roe, C. et al, PLOS Neglected Tropical Diseases| 2022, analyzed ST1005 including the information that there is one virulent isolate, and six attenuated isolates were from the same ST (ST1005). Therefore, suggesting that variably conserved genomic regions may contribute to virulence. The author may discuss and add some information and cite this as a reference.

Minor points

1. The author summary may still over claim in some points such as:
   a. “Molecular typing studies may be utilized to understand the route through which this organism has entered the country”. This may be over elaborated.
   b. “The current study has demonstrated the utility of the multilocus sequence typing scheme for epidemiological studies and clarifies the genetic relationships between *B. pseudomallei* isolated from the clinical specimen and soil samples”. Only 2 soil isolates were analyzed and only one has similar ST with a clinical isolate.
   c. “The findings that the same sequence type present in soil and clinical samples from the same region, will aid in future to track melioidosis outbreaks resulting in transmission breakdown with continued epidemiological monitoring. The study will also play a vital role in establishing a guideline for the prevention and treatment of melioidosis in Bangladesh as well as the basis of vaccine development.” This summary may too far beyond the outcome of this study.
2. Please check some citation for example:
   a. “Strain variation within *B. pseudomallei* is linked to geographic location as well as pattern of land uses [10].” The cited paper compared genes up- or down-expressed in vivo compared to cultures across the three genomes of Bm, Bp, and Bt.
   b. “The variation in clinical manifestations between melioidosis in Australia and Southeast Asia suggests that these populations are genetically distinct due to broad-scale bio-geographical factors associated with the establishment and persistence of the organism [13]” The cited paper reported *B. pseudomallei* in the environment in Puerto Rico.
3. Please clarify the last sentence in the introduction. “We then compared the MLST data with those of the *B. pseudomallei* MLST database to determine the genetic relatedness and attempted to provide an insight into the ecology of *B. pseudomallei* through an elucidation of the mechanisms involved in evolution, speciation, and dispersal.”

4. Lines 148-150: “The YLF and BTFC is a multiplex real-time PCR assay using SYBR green as a fluorescent dye targeting YLF and BTFC gene cluster ….” As YLF and BTFC are gene clusters, mentioned them as a multiplex real-time PCR assay may not appropriate.
5. Abstract line 37: put space between two words “pseudomalleiisolates”
