# Supplementary material for: Phylogeographic characterization of Burkholderia pseudomallei isolated from Bangladesh
Source: PLoS Negl Trop Dis. 2023 Dec 7;17(12):e0011823. doi: 10.1371/journal.pntd.0011823 (PMC10729972; doi:10.1371/journal.pntd.0011823)
Supplement: Attachment — Submitted filename: MLST PNTD REVISED.docx [file pntd.0011823.s010.docx]

Revised MS PNTD-D-22-00909_R1

The revised manuscript title “Phylogeographic characterization of *Burkholderia pseudomallei* isolated from Bangladesh.” was mostly corrected and changed according to what reviewers’ suggestion.

There are a few more comments to be concerned.

As McRobb, E. et al, Applied and Environmental Microbiol, 2014 indicated when analyzed *B. pseudomallei* populations in Australia (779 clinical isolates, including 736 humans plus 43 animal isolates, and 174 environmental strains) using ST analysis that *B. pseudomallei* populations diversified as the sampling area increased. This observation contrasted with smaller sampling areas where a few STs predominated, suggesting that *B. pseudomallei* populations are ecologically established and not frequently dispersed. Therefore, the interpretation using a small number of isolates, even though sending from surrounding areas, in this study should mentioned for their limitation.

The limitation has been included in the discussion (lines 297-301in clean version of the manuscript) as “*The study included small number of isolates from sporadic melioidosis cases, indicating ecologically established population of B. pseudomallei. It contrasts with the study conducted in Australia by Mcrobb et al, which showed diversified population of the bacteria when increased sample size and sampling areas were considered [42]. Further large scale study will help to understand the true ecological establishment of B. pseudomallei in Bangladesh*.”

This manuscript mentioned about ST1005 that isolated from 2 clinical samples and this ST has not been isolated from clinical samples previously from any patients of any country. Roe, C. et al, PLOS Neglected Tropical Diseases| 2022, analyzed ST1005 including the information that there is one virulent isolate, and six attenuated isolates were from the same ST (ST1005). Therefore, suggesting that variably conserved genomic regions may contribute to virulence. The author may discuss and add some information and cite this as a reference.

The comparison of ST 1005 has been included in the discussion (lines 291-296 in clean version of the manuscript) “*A study conducted by Roe et al (2022) analyzed sequence type 1005 isolated from soil. Following inoculation of seven ST 1005 isolates in BALB/c mice, only one was found to be virulent, although it lacked the conserved gene (BPSS0417-BPSS0428) associated with virulence [41]. Analysis of the differences of virulent genes among ST 1005 isolated in different regions of the world will assist in understanding their ability to cause human infection.”*

Minor points

1. The author summary may still over claim in some points such as:
   a. “Molecular typing studies may be utilized to understand the route through which this organism has entered the country”. This may be over elaborated.
   b. “The current study has demonstrated the utility of the multilocus sequence typing scheme for epidemiological studies and clarifies the genetic relationships between *B. pseudomallei* isolated from the clinical specimen and soil samples”. Only 2 soil isolates were analyzed and only one has similar ST with a clinical isolate.
   c. “The findings that the same sequence type present in soil and clinical samples from the same region, will aid in future to track melioidosis outbreaks resulting in transmission breakdown with continued epidemiological monitoring. The study will also play a vital role in establishing a guideline for the prevention and treatment of melioidosis in Bangladesh as well as the basis of vaccine development.” This summary may too far beyond the outcome of this study.

The author summary has been corrected as follows:

*Melioidosis, caused by Burkholderia pseudomallei, is a disease of various manifestations and poor outcome, if not diagnosed and treated early. The first case of melioidosis is believed to be identified from Bangladesh as early as 1960, although the persistence of the causative organism was discovered in soil about 50 years later, in 2012. The study utilized 20 B. pseudomallei isolated from clinical samples and 2 from soil samples. Multi Locus Sequence Typing (MLST) was conducted with all 22 isolates and the sequence types analyzed and compared with B. pseudomallei sequence types previously detected from other countries. Four novel sequence types (ST) were detected in our study. Out of 12 different STs determined, ST 1005, 1007 and 56 were the commonest sequence types identified in Bangladesh. ST 1005 was found in both clinical and soil samples. ST 56 was isolated from five septicemic patients. The study revealed the sequence types of B. pseudomallei isolated from Bangladesh and their genetic relatedness with sequence types of other regions. We further showed that the sequence types of Bangladesh share more genetic similarities with the isolates of Southeast Asian isolates rather than South Asian isolates.*

2. Please check some citation for example:
   a. “Strain variation within *B. pseudomallei* is linked to geographic location as well as pattern of land uses [10].” The cited paper compared genes up- or down-expressed in vivo compared to cultures across the three genomes of Bm, Bp, and Bt.

Reference-10 has been changed.

[10] Pearson TR, Price EP, Tuanyok A, Keim P. Genetic diversity and geographic distribution of Burkholderia pseudomallei. In: Keethsan N, editor. Melioidosis: a century of observation and research. Amsterdam: Elsevier B.V.; 2012. pp. 340-348.

b. “The variation in clinical manifestations between melioidosis in Australia and Southeast Asia suggests that these populations are genetically distinct due to broad-scale bio-geographical factors associated with the establishment and persistence of the organism [13]” The cited paper reported *B. pseudomallei* in the environment in Puerto Rico.

The reference has been changed to [11].

[11] Tuanyok A, Auerbach RK, Brettin TS, Bruce DC, Munk AC, Detter JC, et al. A horizontal gene transfer event defines two distinct groups within Burkholderia pseudomallei that have dissimilar geographic distributions. *Journal of bacteriology*. 2007; **189**(24): 9044–9.

All references have been rearranged accordingly.

3. Please clarify the last sentence in the introduction. “We then compared the MLST data with those of the *B. pseudomallei* MLST database to determine the genetic relatedness and attempted to provide an insight into the ecology of *B. pseudomallei* through an elucidation of the mechanisms involved in evolution, speciation, and dispersal.”

*Has been rephrased as “To explore the genetic relatedness of our local isolates with* isolates detected previously from other countries*, we collected and compared sequences from PubMLST database [22].”*

4. Lines 148-150: “The YLF and BTFC is a multiplex real-time PCR assay using SYBR green as a fluorescent dye targeting YLF and BTFC gene cluster ….” As YLF and BTFC are gene clusters, mentioned them as a multiplex real-time PCR assay may not appropriate.

*Has been rephrased as “The YLF and BTFC gene clusters were detected using real-time PCR assay developed by Tuanyok A et al in 2007.”*

5. Abstract line 37: put space between two words “pseudomalleiisolates”- *has been corrected.*

Figure Files: All figures have been uploaded to PACE tool and the converted files have been uploaded.

Data requirements: All available data has been included in the supplementary file.
